# *RNxQuest*: An Extension to the *xQuest* Pipeline Enabling Analysis of Protein–RNA Cross-Linking/Mass Spectrometry Data

**DOI:** 10.1021/acs.jproteome.3c00341

**Published:** 2023-09-05

**Authors:** Chris P. Sarnowski, Michael Götze, Alexander Leitner

**Affiliations:** † Institute of Molecular Systems Biology, Department of Biology, ETH Zürich, 8093 Zurich, Switzerland; ‡ Systems Biology PhD Program, University of Zürich and ETH Zürich, 8093 Zurich, Switzerland

**Keywords:** cross-linking mass spectrometry, protein−RNA interactions, ribonucleoproteins, XL-MS, XL-MS software, false discovery rate estimation

## Abstract

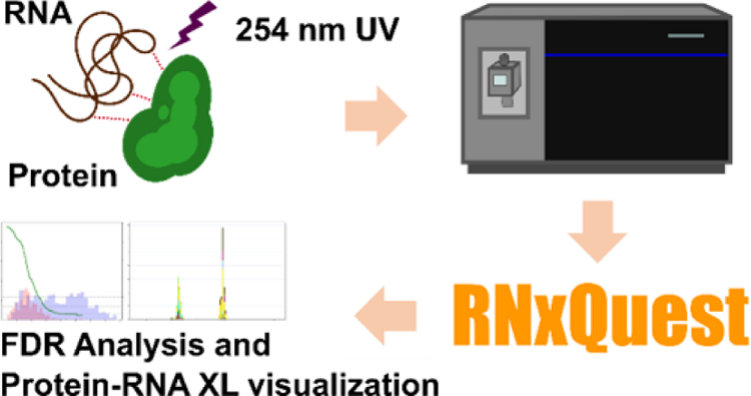

Cross-linking and mass spectrometry (XL-MS) workflows are increasingly popular techniques for generating low-resolution structural information about interacting biomolecules. *xQuest* is an established software package for analysis of protein–protein XL-MS data, supporting stable isotope-labeled cross-linking reagents. Resultant paired peaks in mass spectra aid sensitivity and specificity of data analysis. The recently developed cross-linking of isotope-labeled RNA and mass spectrometry (CLIR-MS) approach extends the XL-MS concept to protein–RNA interactions, also employing isotope-labeled cross-link (XL) species to facilitate data analysis. Data from CLIR-MS experiments are broadly compatible with core *xQuest* functionality, but the required analysis approach for this novel data type presents several technical challenges not optimally served by the original *xQuest* package. Here we introduce *RNxQuest*, a Python package extension for *xQuest*, which automates the analysis approach required for CLIR-MS data, providing bespoke, state-of-the-art processing and visualization functionality for this novel data type. Using functions included with *RNxQuest*, we evaluate three false discovery rate control approaches for CLIR-MS data. We demonstrate the versatility of the *RNxQuest*-enabled data analysis pipeline by also reanalyzing published protein–RNA XL-MS data sets that lack isotope-labeled RNA. This study demonstrates that *RNxQuest* provides a sensitive and specific data analysis pipeline for detection of isotope-labeled XLs in protein–RNA XL-MS experiments.

## Introduction

Cross-linking coupled to mass spectrometry (XL-MS) is a popular approach for obtaining structural information from molecular complexes. Structural information is obtained in a protein–protein XL-MS workflow by covalent cross-linking of spatially proximal amino acids of proteins or protein complexes in solution. Samples are then prepared for analysis with liquid chromatography (LC) coupled to tandem mass spectrometry (MS/MS) using a modified bottom-up proteomics workflow. Through amino-acid specific chemical reactivity of cross-linking reagents and/or peptide sequencing by MS/MS, precise cross-linked amino acid positions are identified in a pair of peptides that must be in close proximity within protein structures in their native state.^1−3^ These proximal amino acid positions are frequently used to define distance restraints in structural modeling pipelines,^4,5^ and prove particularly useful for flexible complexes, where individual molecules do not conform to a homogeneous structural state upon crystallization.

Proteins do not just form complexes with other proteins, but commonly interact with nucleic acids such as RNA, either transiently, such as for regulatory functions, or in forming stable protein–nucleic acid complexes that function as vital catalytic cellular machinery.^6,7^ As with protein–protein cross-links (XLs), protein–RNA XLs derived from mass spectrometric workflows also form a useful input data type for structural modeling pipelines.^8−10^ However, their use is currently less widespread than protein–protein cross-linking, and there are fewer dedicated software solutions for this data type. Most protein–RNA XL-MS workflows developed so far rely on photochemical cross-linking, where protein–RNA complexes are irradiated under UV light. However, proteomics data sets produced using this approach are particularly challenging to analyze.

The UV cross-linking reaction tends to be inefficient, with low proportions of starting material converted to cross-linked complex^11^ and low numbers of XL spectrum matches (XLSMs) during data analysis. To overcome this challenge, the data analysis approach therefore needs to be both sensitive enough to make identifications close to the detection limit but also specific enough to avoid false positive identifications, the prevalence of which can be challenging to assess where low numbers of identifications make it difficult to achieve statistical robustness.

When purified protein–RNA complexes are analyzed, previously published approaches propose a variety of strategies to overcome these challenges. According to best practice, the RNP^xl^ workflow^8,12^ should be executed with the input of both UV cross-linked and non-cross-linked control samples for data analysis (although it is also technically possible to run the pipeline without a control sample). This comparative approach ensures that low intensity and abundance peptide modification signals present in the UV irradiated sample are absent in the control sample and therefore likely derive from cross-linked RNA. However, the addition of a negative control data set doubles both sample requirement and associated mass spectrometric analysis time, making this a resource intensive solution. Furthermore, analysis of low abundant protein–RNA XL species in a complex background may result in undersampling of MS/MS precursors, resulting in reduced sensitivity in XL detection.

An alternative approach is proposed in the RBS-ID workflow,^13^ which uses chemical degradation of RNA in place of more widely adopted nuclease digestion, to achieve homogeneous mononucleotide modifications on peptides. This results in a relative signal boost by avoiding the stratification of cross-linked species between heterogeneous polynucleotide adducts attached at a given amino acid site on a peptide.^14^ Given the very limited number of peptide modifications provided, data can then be analyzed as with post-translational modifications, using either open search engines^15,16^ or more conventional closed search approaches.^17,18^ While this approach is very effective at identifying protein sites that are in contact with RNA, the absence of polynucleotide adducts in analysis results makes assignment of the cross-linked nucleotide within an RNA sequence more difficult. This is therefore a less suitable approach for derivation of distance restraints for structural modeling of protein–RNA complexes,^14^ where precise placement of a XL is required on both RNA and protein sequences.

The recently introduced CLIR-MS approach^9^ utilizes stable isotope labeling of RNA to overcome the challenges posed by data collected from UV cross-linked complexes. In a CLIR-MS experiment, both light and heavy isotopic forms of a given RNA-derived peptide modification must be detected during data analysis in order to produce an identification, ensuring peptide modifications truly derive from RNA. Furthermore, when utilizing the popular *xQuest* search engine, increased sensitivity is achieved during analysis by merging light and heavy spectra as described previously.^19,20^ The distinguishing advantage of the CLIR-MS approach is the ability to incorporate stretches of stable isotope-labeled RNA in a position specific fashion, as demonstrated previously,^9,14,21,22^ therefore providing robust localization of the XL in an RNA sequence, as required for definition of distance restraints for structural modeling of protein–RNA complexes.

In order to realize fully the advantages provided by the CLIR-MS approach, a specialized data analysis approach based on the *xQuest* search engine^19^ was established. This approach uses multiple parallel searches, one per expected delta mass shift (expected mass difference between the light and heavy forms) for isotope-labeled RNA adducts in the sample.^9^ The analysis approach is flexible and can be adapted to different delta mass shifts introduced by use of different RNA labeling strategies, for example through ^13^C^15^N metabolic labeling,^9^ through use of ^13^C ribose,^21^ or through appendage of an ^18^O phosphate group.^22^ When one attempts to use just the base *xQuest* functionality alone for this type of search strategy, it is practically prohibitive to manually define the required parameter sets for each of these searches. Furthermore, the requirement for multiple parallel *xQuest* searches, with the exact number varying depending on RNA labeling strategy, introduces a technical incompatibility with the false discovery rate (FDR) control companion software, *xProphet*,^20^ leaving protein–RNA cross-linking identifications reliant on manual validation, or restricted to arbitrary score cut-offs for quality control.

Here we introduce *RNxQuest*, a state-of-the-art Python package companion to the established *xQuest* pipeline. The package provides a number of features to support the analysis of protein–RNA XL-MS data produced using the CLIR-MS technique and, to our knowledge, is the only package specifically designed to handle this data type. The package is primarily designed for analysis of low complexity protein–RNA samples prepared with stable isotope-labeled RNA. With the help of *RNxQuest*, parameter generation and execution for the required parallel searches are automated, simplifying data analysis. Furthermore, the package provides FDR control functionality, overcoming the incompatibility of *xProphet* with protein–RNA cross-linking data. We take advantage of recent advances in the method which improve the number of XLSMs,^14^ and investigate the effectiveness of different FDR control strategies for obtaining unique structural information from CLIR-MS data sets. Finally, the *RNxQuest* package provides a postprocessing pipeline in the form of a JuPyter notebook that facilitates further filtering (i.e., by mass error) and visualization. We additionally compare the outputs of the overall search strategy and postprocessing pipeline enabled by the novel *RNxQuest* functionality with approaches used to analyze other recently published protein–RNA XL-MS data sets, which employ non-isotope-labeled RNA. Together, we demonstrate that *RNxQuest* excels in its primary function as a dedicated software solution for CLIR-MS data, while maintaining broad technical applicability, even for data sets without isotope-labeled RNA.

## Experimental Section

### Preparation of a Heterogeneous Isotope-Labeled Monolink Sample Using BSA

100 μg aliquots of 5 mg/mL bovine serum albumin (BSA, Sigma-Aldrich) was prepared in 1× PBS. Eight aliquots were prepared in total so that each cross-linking reagent type could be prepared in duplicate. Cross-linking with DSS and DSG was carried out as described previously.^23^ Cross-linking stock solutions for DSS (equimolar mixture of d_0_ and d_12_ isotope-labeled forms, Creative Molecules) and DSG (equimolar mixture of d_0_ and d_6_ isotope-labeled forms, Creative Molecules) were freshly prepared in anhydrous dimethylformamide at a concentration of 25 mM. Cross-linking reactions took place with final protein concentration at 0.67 mg/mL and final cross-linker concentration of 0.5 mM in 20 mM HEPES (4-(2-hydroxyethyl)-1-piperazineethanesulfonic acid). A shortened incubation time was used to favor monolink formation (20 min, 37 °C). All incubations below were carried out using a ThermoMixer (Eppendorf, 800 rpm). Reactions were quenched by addition of ammonium bicarbonate (AmBic) to a final concentration of 50 mM, followed by a further incubation (20 min, 37 °C). Cross-linking with ADH and PDH was carried out as described previously.^24^ Stock solutions were prepared for ADH (equimolar mixture of ADH-d_0_ and -d_8_, Sigma-Aldrich), PDH (equimolar mixture of PDH-d_0_ and -d_10_, Sigma-Aldrich) and DMTMM (4-(4,6-dimethoxy-1,3,5-triazin-2-yl)-4-methyl-morpholinium chloride, Sigma-Aldrich), all at approximately 100 mg/mL in 20 mM HEPES. For ADH, cross-linking reactions took place with a protein concentration of 0.67 mg/mL, and final concentrations of 8.3 and 12 mg/mL for ADH (sum of both isotope forms) and DMTMM, respectively, in 20 mM HEPES. For PDH, concentrations were identical except adjustments for its different relative mass (sum of both isotope forms). As with DSS and DSG, a shortened incubation time was used to favor monolink formation (15 min, 37 °C). Reactions were quenched by gel filtration using Zeba spin columns (Thermo Scientific).

After quenching of cross-linking reactions, all samples were dried in a vacuum centrifuge, and prepared for LC-MS/MS analysis following the same established protocol.^24^ Samples were resuspended in 8 M urea to a final protein concentration of 1 mg/mL. Disulfide bonds were reduced by addition of tris(2-carboxyethyl) phosphine to a final concentration of 2.5 mM followed by incubation (30 min, 37 °C). Free cysteines were then alkylated by the addition of iodoacetamide to a final concentration of 5 mM, followed by incubation (30 min, 25 °C, in the dark). The urea concentration was adjusted to 6 M by addition of a 1 M AmBic stock solution, Lys-C (Wako Chemicals) was added at 1:100 enzyme:substrate ratio, and samples were incubated (3 h, 37 °C). After Lys-C digestion, the urea concentration was further adjusted to 1 M by addition of a 1 M AmBic stock solution, and trypsin (Promega) was added at a 1:24 enzyme:substrate ratio, in the presence of ProteaseMax (Promega) rapid digestion surfactant according to the manufacturer’s instructions. Samples were once again incubated (3 h at 37 °C). Following digestion, proteases were quenched by addition of formic acid (FA) to 2% of total volume, and samples were cleaned up by C_18_ solid phase extraction (50-mg SepPak tC18 cartridges, Waters). Eluents were dried in a vacuum centrifuge. Samples from the four different cross-linking reagents were then pooled into a single tube, which was then prepared for peptide-level size exclusion chromatography (SEC) fractionation as described previously,^23−25^ using a Superdex Peptide PC 3.2/30 column (GE Healthcare). Four 100 μL fractions, expected to contain the majority of monolinked peptides, were selected, corresponding to elution volumes of 0.7 to 1.1 mL, for analysis by LC-MS/MS.

### Analysis of Heterogeneous Monolink Samples by LC-MS/MS

Individual SEC fractions were evaporated to dryness in a vacuum centrifuge and resuspended in MS mobile phase A (described below) to a concentration of 0.5 mg/mL according to the UV signal intensity from the SEC chromatograms at the time of fraction collection. 2 μL of each sample was injected, and each sample was injected twice. LC-MS/MS analysis was carried out using an Easy nLC 1000 HPLC system (ThermoFisher Scientific) coupled to an Orbitrap Elite mass spectrometer (ThermoFisher Scientific), using a Nanoflex electrospray ion source. Samples were separated on a PepMap RSLC column (150 mm × 75 μm, 2 μm particle size, ThermoFisher Scientific) with gradient of 9 to 35% mobile phase B over 60 min (A = water:acetonitrile:FA, 98:2:0.15, v/v/v; B = acetonitrile:water:FA, 98:2:0.15, v/v/v). The flow rate used for analysis was 300 nL/min. The data-dependent acquisition mode was used for the mass spectrometer. The Orbitrap mass analyzer was used for precursor ion spectra acquisition at a resolution of 120,000. For each precursor acquisition cycle, the top 10 ions were selected for collision-induced dissociation fragmentation, and fragment ions were analyzed in the linear ion trap by using the normal scan rate. Further fragmentation parameters used for the analysis were the following: isolation width, 2 *m*/*z*; normalized collision energy, 35; activation time, 10 ms; dynamic exclusion for 30 s after one sequencing event was enabled. All files were converted from Thermo raw format into mzXML using the msConvert^26^ package.

### Data Analysis for Heterogeneous Monolink Samples

Converted mzXML files were searched with *xQuest*(19,20) (version 2.1.5, available at https://gitlab.ethz.ch/leitner_lab/xQuest_xprophet) against a target and decoy database for bovine serum albumin protein. The decoy database was generated using the xdecoy.pl script included with *xQuest*, using the reverse and shuffle features sequentially. Individual analysis runs for each data set were defined manually (without the *RNxQuest* package), one per expected light-heavy delta mass in the data set (i.e., one per cross-linking reagent). A mass tolerance of 10 ppm for the mass shift was used, with a maximum retention time difference between scan pairs of 1 min. Additional search settings: enzyme, trypsin; maximum missed cleavages, 2; MS mass tolerance, 10 ppm; MS/MS mass tolerance, 0.2 Da for common ions and 0.3 Da for XL ions. Only monolink type identifications were searched for. Amino acid reactivity was restricted to the known reactivity of the cross-linking reagents (lysine for DSS and DSG, aspartic acid and glutamic acid for ADH and PDH). Where FDR analysis was carried out using *xProphet*(20) (version 2.5.5, available at https://gitlab.ethz.ch/leitner_lab/xQuest_xprophet), default parameters were used. Outputs are listed in Table S1.

### The RNxQuest Python Package

Scripts are provided by the *RNxQuest* package to facilitate automated parallel *xQuest* searches for the CLIR-MS data. The package source code is publicly available (https://gitlab.ethz.ch/leitner_lab/RNxQuest). Installation instructions, usage tutorials, and extensive documentation of functions included in the package are available in the associated repository wiki site (https://gitlab.ethz.ch/leitner_lab/rnxquest/-/wikis/home). The package is designed to function in Windows or Unix-like environments, using Python 3.7.1, and with desktop grade hardware. *RNxQuest* depends on the following Python packages: lxml, pandas, fastaparser, matplotlib, numpy, plotly, scipy, pathlib, mokapot. The first step in the *RNxQuest* analysis pipeline is automated parameter generation. Using a uniform set of input files and sample-related parameters (databases, mzXML files, RNA sequence, template *xQuest* definition files), a folder structure, customized parameter files, and Unix shell scripts are generated that facilitate automated search execution for the parallel searches required for a CLIR-MS data set. After search execution, the *RNxQuest* package provides functions to extract and consolidate the results from all parallel searches into a single file, to facilitate further downstream analysis, while additionally merging the results into groups that are backward compatible with the *xQuest* result/spectrum viewer.^19^

The package also includes a template postprocessing analysis pipeline in the form of a JuPyter Notebook.^27^ The standard pipeline provides for FDR analysis and subsequent filtering of results according to the desired threshold. A function is also provided to refine results by mass error, fitting the observed relative mass errors for all target identifications to a curve, and using it to define an acceptable error tolerance that reflects the data set. The *RNxQuest* parallel search approach requires reranking of identifications, to ensure that the same spectrum is not used in identifications produced by each of the parallel *xQuest* searches—a function is provided in the package for this purpose. The available FDR control approaches and filtering steps are further illustrated in Figure S1. The package and JuPyter notebook also contain two suggested methods of visualizing the data produced in a CLIR-MS experiment (Figure S2). The first creates a stacked bar plot of RNA sequences detected at a given amino acid composition, to give a protein-centric view of observed XLs. Alternatively, these observed RNA adducts can be compared and overlaid upon the RNA sequence used to prepare the sample, to create a probabilistic representation of the likely interaction site on the RNA. The latter approach is most useful when used with a short, nonredundant labeled RNA sequence. Finally, the JuPyter notebook outputs a summary file in .csv format, containing the suggested minimum information required for further use of the data in a structural modeling pipeline, or for reporting results.

### Data Analysis for Model Protein-RNA Complexes with RNxQuest

The sample preparation procedure for these complexes is described elsewhere.^14^ The raw files were converted to mzXML format using msConvert.^26^ Parameters were generated for searching each sample using the *RNxQuest* parameter generation script. The protein database used for each search consisted of the target sequence for the respective protein and a reversed and shuffled decoy database sequence generated using the *xQuest* function xdecoy.pl (described above). Input RNA sequences correspond to the labeled RNAs used to prepare the samples^14^—UGCAUGU, CGCUU, UCUCU for the FOX1, MBNL1, and PTBP1 complexes, respectively. The default *RNxQuest* definition files were used as a basis for the search; these are described in more detail in the documentation for the package (link above). Mass tolerances were set in the same way as for the heterogeneous monolink sample (described above). Searches were executed, and results were consolidated using parameter generation, merging, and extraction functionality provided by the *RNxQuest* package.

### FDR Analysis Using RNxQuest Observed FDR Calculation, Transferred FDR, and mokapot

The default FDR function provided in the *RNxQuest* package uses the relative proportions of target and decoy database identifications above a given ld-score threshold to determine the false discovery rate, and is a technical reimplementation of the calculation described for the monolink group in *xProphet*.^20^ Where this function is used, calculations are made at the unique identification level and results are filtered with an FDR of less than 1%. The 1% value was selected for sufficient stringency and is the default value suggested to users. However, this can be adjusted by the user. Here, an identification is defined as a unique combination of the amino acid sequence position and cross-linked RNA product.

An alternative FDR analysis approach using the *mokapot* package is also provided as a function in *RNxQuest*. The conversion function included with the *RNxQuest* package takes the consolidated *RNxQuest* output csv file, and reformats it into a PIN file, compatible with the *Percolator* algorithm^28^ and the *mokapot* package.^29^ The *xQuest* subscores used to calculate the ld-score, plus some additional RNA-specific features such as the calculated peptide mass addition, are outputted as features by the *RNxQuest* PIN conversion function. The full list of features in the PIN output file is listed in Table S2. Where identifications are analyzed using the *mokapot* FDR analysis approach, the analysis is executed at the peptide/identification level (according to the definition above), and resulting identifications are filtered for those with a q-value below 0.01.

The transferred FDR calculation is implemented as an *RNxQuest* function as described previously.^30,31^ For analyses described here, identifications are binned according to the sequence composition of the RNA component of the identification. We reason that this most closely reflects the biological question in a CLIR-MS experiment (i.e., which RNA sequence is in contact with a given amino acid in a protein–RNA complex). The lowest 80% of unique decoy identification score values is used for linear approximation in all cases, to avoid the issue of lower counts of decoy identifications at higher score thresholds described previously.^30^ An identification level transferred FDR was calculated for each bin by using the function in the *RNxQuest* package. An ld-score threshold at the specified transferred FDR threshold (<1%) is returned for each bin, facilitating XLSM filtering. Given the low numbers of overall identifications expected in a CLIR-MS experiment, bins with insufficient numbers of decoy identifications for a mathematically valid calculation are expected frequently. To account for this, a backup strategy is implemented to use the FDR computed for the complete data set in such cases, using the *RNxQuest* observed FDR calculation instead. After FDR analysis, XLSMs are filtered according to a 1% FDR threshold.

After all types of FDR analysis, postprocessing steps provided in the default JuPyter notebook described above were followed to further refine the list of identifications, before comparing outputs of each strategy. Lists of unique amino acid positions and RNA adduct masses were used as a basis for comparison. Outputted XLSMs from each FDR approach are provided in Tables S3, S4, and S5 for the FOX1, MBNL1, and PTBP1 protein–RNA XL-MS data sets, respectively.

### Reanalysis of Previously Published Data Sets

For the previously published human RBP complex data,^8^ corresponding raw mass spectrometry files were downloaded from the PRIDE repository (data set identifier: PXD000513). For the Cas9 protein cross-linked to an unlabeled sgRNA,^13^ corresponding raw mass spectrometry files were also downloaded from the PRIDE repository (data set identifier: PXD016254). Files were converted to the mzXML format using msConvert.^26^ Parameters were generated for searching each sample using the *RNxQuest* parameter generation script. The protein database used for each search consisted of the target sequences for the respective proteins in the sample plus reversed and shuffled decoy database sequences, as described above. For the human RNA binding protein data, a theoretical RNA sequence computed to contain all possible combinations of all mono-, di-, tri-, and tetranucleotides (given 4 bases) was used to generate parameters (AAAAAAACAAAGAAAUAACCAACGAACUAAGGAAGUAAUUACCCACCGACCUACGGACGUACUUAGGGAGGUAGUUAUUUCCCCCCCGCCCUCCGGCCGUCCUUCGGGCGGUCGUUCUUUGGGGGGGUGGUUGUUUUUUU). For the Cas9 sample, the reported sgRNA sequence^13^ was used for search parameter generation (GGCGCAUAAAGAUGAGACGCGUUUUAGAGCUAGAAAUAGCAAGUUAAAAUAAGGCUAGUCCGUUAUCAACUUGAAAAAGUGUUCG). The default *RNxQuest* definition files were used as a basis for the search—these are described in more detail in the documentation for the package (link above). Mass tolerances were defined in the same way as for the heterogeneous monolink sample (described above). Searches were executed, and results consolidated using parameter generation, merging, and extraction functionality provided by the *RNxQuest* package. FDR analysis was carried out using the *RNxQuest* observed FDR calculation, and identifications with an ld-score where the FDR was below 1% were retained. Further postprocessing was carried out using the *RNxQuest* JuPyter notebook as described above. The number of XLSMs produced by the *RNxQuest* pipeline was then compared with the reported XLSMs with which the data sets were published. Outputs from the reanalysis of Cas9 and Human RBP data sets with *RNxQuest* are included in Table S6.

### Validation of Protein-RNA Cross-Links Identified with RNxQuest

For the model complexes, FOX1, MBNL1, and PTBP1, protein–RNA XL distances were compared with a list of distances measured in a previous report.^14^ For peptide–RNA combinations for which no prior measurement was available due to detection of a novel RNA composition, the nearest mononucleotide distance to a previously measured XL with matching peptide sequence was used. Where a novel cross-linked peptide species was detected compared with the previous data, the nearest single nucleotide of any compatible with the detected RNA composition was used for the measurement. For the Cas9 complex, peptide–RNA XLs with nonmononucleotide-U RNA composition were considered for validation against a published structure (PDB 4ZT0)^32^ due to complementarity to previously published identifications. Validation of the RNA component was not, in the absence of segmentally isotope-labeled RNA, considered due to the much increased length of the RNA in the structure compared with the FOX1, MBNL1, and PTBP1 complexes, and the redundant nature of nucleic acid sequences. Validation of amino acid positions was undertaken by inspection of the proximity to the RNA chain in the published structure. For all complexes mentioned above, in a small minority of cases, it was not possible to validate novel cross-links due to the sequence used for protein–RNA XL-MS experiments diverging from that in the structure (i.e., truncation, differences in purification tag). Representative annotated spectra for the FOX1 and Cas9 complexes are provided in the Supporting Information, and were annotated using the Interactive Peptide Spectral Annotator.

### Data Availability

All *RNxQuest* outputs have been deposited to the ProteomeXchange Consortium (http://proteomecentral.proteomexchange.org) via the PRIDE^34^ partner repository with the data set identifier PXD039754. Raw data for the model protein–RNA complexes have been deposited to the same repository with data set identifier PXD029930. Raw data for the Cas9 and human RBP complex data sets were downloaded from the PRIDE repository using data set identifiers PXD016254 and PXD000513, respectively.

### Software Availability

The *RNxQuest* package source code is available for download from a public repository (https://gitlab.ethz.ch/leitner_lab/RNxQuest), along with extensive documentation in the associated repository wiki site (https://gitlab.ethz.ch/leitner_lab/rnxquest/-/wikis/home).

## Results

### Distinct xQuest Configuration for CLIR-MS Protein-RNA Cross-Linking Data

**Figure 1:**
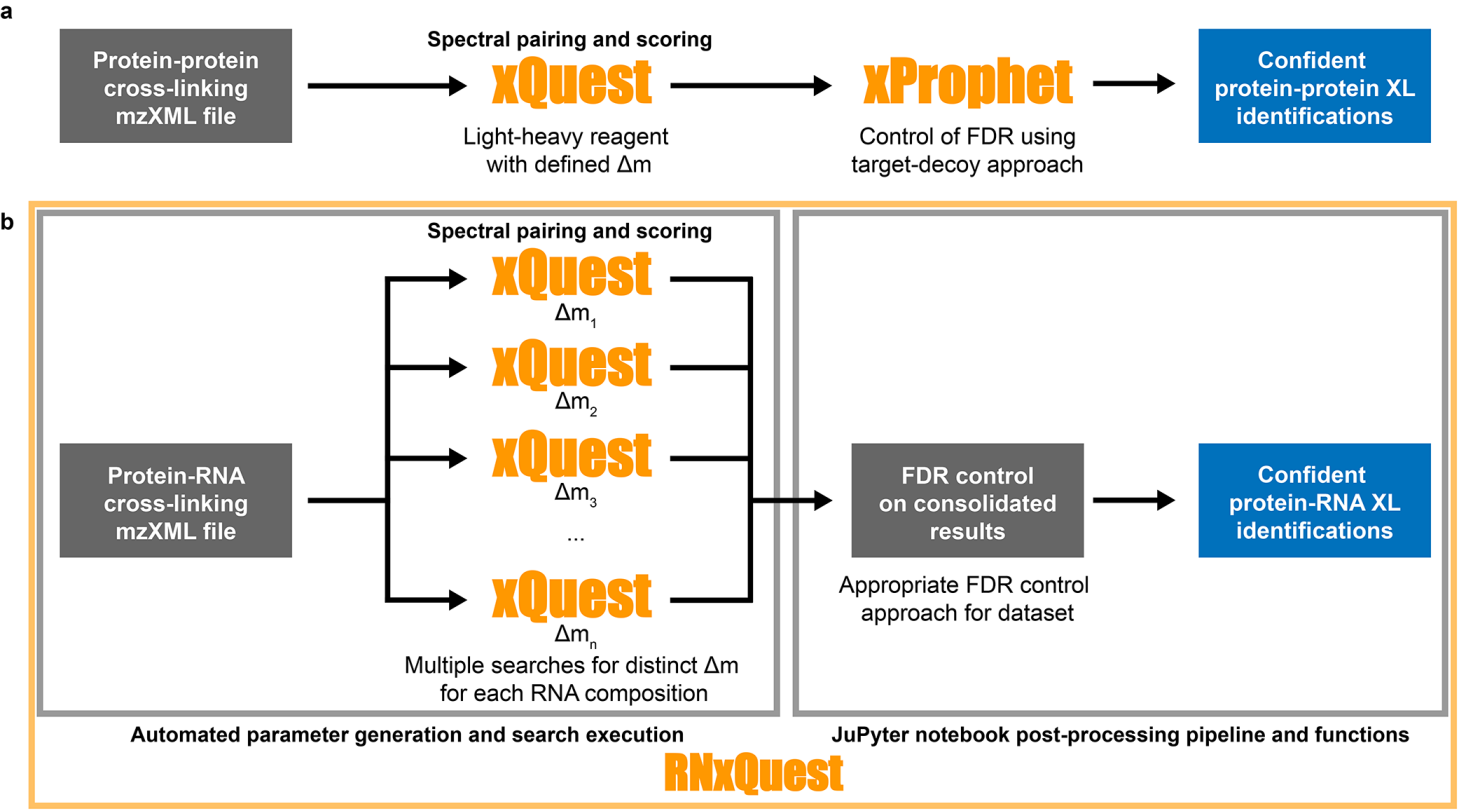
Schematic comparison of *xQuest* search approaches for protein–protein and protein–RNA XL-MS data sets. (a) Schematic representation of the *xQuest* analysis pipeline for a conventional protein–protein XL-MS workflow. (b) Schematic representation of the *xQuest* analysis pipeline for a protein–RNA XL-MS workflow using stable isotope-labeled RNA. Two broad groups of functionality enabled by the package are highlighted in gray boxes: automated parameter generation with search execution, and FDR control postprocessing and visualization with a JuPyter notebook.

The *xQuest* software package was originally designed for analysis of protein–protein XL-MS data, especially for supporting experiments that employ a light-heavy isotope paired cross-linking reagent. Therefore, when defining an *xQuest* search, one of the key parameters is the delta mass of the cross-linked species. In routine protein–protein XL-MS experiments, a single cross-linking reagent is added to the sample, resulting in a single expected delta mass. Such data can therefore be analyzed with a single *xQuest* search, as schematically illustrated in Figure 1a. In the case of protein–RNA XL-MS experiments using the CLIR-MS technique,^9^ we propose that light-heavy isotope paired RNA modifications are conceptually similar to protein–protein XL-MS monolinks (also known as dead-end links). However, many different lengths and sequence compositions of RNA products are expected to be attached to peptides in the same sample, resulting from nuclease digestion (for example mono-, di-, tri-, and tetranucleotides). These can also exhibit many different delta masses, depending on the RNA labeling strategy used. Therefore, RNA species cross-linked to peptides in a CLIR-MS sample resemble a set of heterogeneous monolink species in a protein–protein XL-MS sample that has been prepared with several different isotope-labeled cross-linking reagents. With multiple delta masses expected in the sample, multiple parallel *xQuest* searches of the data are required to discover all species expected in a sample, illustrated in Figure 1b. The *RNxQuest* Python package provides two broad groups of functions, highlighted in Figure 1b, to facilitate the parallel analyses required when searching CLIR-MS data using *xQuest*.

First, the *RNxQuest* package provides automation for parameter file generation, search execution, and result consolidation required for the parallel search strategy (Figure 1b, left box). A parameter generation script takes account of the RNA sequence provided, which RNA isotope labeling strategy was used for sample preparation, and (optional) grouping of input mzXML files by sample type. Based on these, a set of *xQuest* search parameter files are generated, together containing all possible RNA-derived modifications that can occur based on the input nucleotide sequence, grouped into parallel searches based on the expected delta masses. RNA-derived products specified at this point can also include the many neutral loss products thought to arise from the nucleic acid moiety.^14^ Additionally, Unix shell scripts are provided which automatically execute these searches and consolidate their results in a single output file. Separately, compatibility with the *xQuest* viewer^19^ is also maintained for analysis of spectra. As a result, a comprehensive CLIR-MS data search can be undertaken in as few as three commands, eliminating the practical inconvenience of defining and executing each manually, as was previously necessary.^9^

Second, the *RNxQuest* package provides bespoke postprocessing functionality required for analysis of CLIR-MS data (Figure 1b, right box). Consolidated *xQuest* results from multiple searches of the same data set require reranking of identifications made from each spectrum to ensure the same scan is not assigned multiple times. A function is provided for this. Furthermore, *RNxQuest* provides tools to further refine identifications, such as by mass error, and for visualization of CLIR-MS XLSMs. *RNxQuest* also provides solutions to overcome the inherent incompatibility of CLIR-MS data with the protein–protein XL-MS FDR estimation package *xProphet*.^20^ From a technical standpoint, when *xQuest* was used according to the scheme in Figure 1b, *xProphet* would need to be executed once per parallel search. However, given the sparseness of enriched protein–RNA XL-MS samples, each search in isolation is expected to only produce a small number of XLSMs, upon which it is difficult to reliably estimate the FDR.^35^ To achieve more accurate estimations, we therefore propose FDR estimation based on the consolidated identifications from the parallel searches instead, for which multiple functions are provided in *RNxQuest*, supporting several distinct FDR control approaches (see below and Figure S1). Together, the tools in the *RNxQuest* package facilitate complete analysis of CLIR-MS data, from the mzXML file to data visualization. Generic examples of the output provided by the postprocessing pipeline are shown in Figure S2.

As an initial approach for FDR estimation in CLIR-MS data, we reimplemented the target-decoy competition (TDC) calculation used by *xProphet*(20) as a Python-based function, included in the *RNxQuest* package. This is referred to as the “*RNxQuest* observed FDR calculation” from here onward.

### Benchmarking the Performance of the Novel RNxQuest Configuration

**Figure 2:**
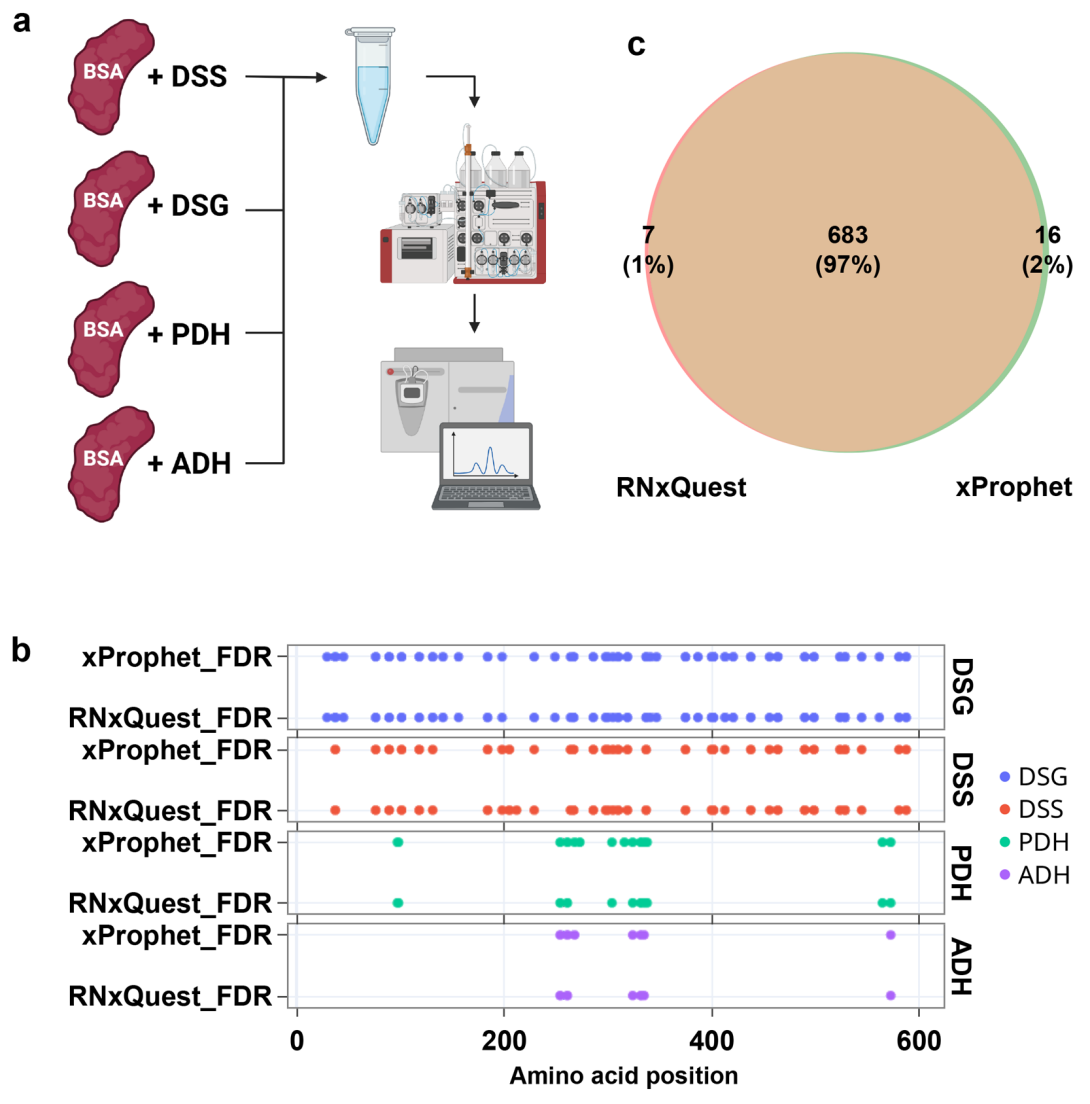
Benchmarking the performance of the *RNxQuest* observed FDR calculation with a heterogeneous protein–protein XL-MS monolink data set. (a) Schematic representation of experimental steps to produce the heterogeneous monolink data set. This panel was created with BioRender.com. (b) Monolinks identified on BSA for each of the cross-linkers, where FDR is controlled either by *xProphet* at the end of each *xQuest* run for each Δm or using a combined observed FDR calculation on the complete set of identifications. (c) Overlap of XLSMs when FDR is controlled using *xProphet* after each single run or using the observed FDR calculation calculated on the complete set of identifications.

We first compared the reimplemented observed FDR calculation in *RNxQuest* with the original implementation in *xProphet*, to ensure faithful reproduction of the behavior of *xProphet*. To undertake this comparison, we required a data set compatible with both approaches. We therefore cross-linked bovine serum albumin (BSA) protein with four different light-heavy isotope paired protein–protein cross-linking reagents: disuccinimidyl suberate (DSS), disuccinimidyl glutarate (DSG), adipic acid dihydrazide (ADH), and pimelic acid dihydrazide (PDH). Samples were then pooled after the respective cross-linking reactions were quenched, and pooled samples digested and fractionated by size exclusion chromatography as described previously.^23^ Fractions expected to contain mostly monolink modifications were selected for analysis by LC-MS/MS. The experiment is summarized in Figure 2a. The result is a data set that contains many heterogeneous isotope paired peptide modifications. These mimic the properties of heterogeneous RNA adducts found in a CLIR-MS protein–RNA cross-linking experiment, requiring multiple parallel *xQuest* analyses to discover all monolink types present in the data set. Additionally, owing to the higher expected yields of these reactions compared with a protein–RNA UV cross-linking reaction, sufficient numbers of unique identifications for each monolink type are expected that *xProphet*-based FDR analysis is still able to estimate FDR in each parallel run. Data were analyzed using four manually defined parallel *xQuest* searches, one per distinct expected delta mass between the light and heavy isotopic forms of the monolinks, according to the approach shown in Figure 1b, thereby mimicking the required approach for CLIR-MS data.

FDR analysis was then carried out twice for the data set: once using four parallel *xProphet* executions, one per search for each expected delta mass (as supported by core *xQuest*/*xProphet* functionality), and once using the observed FDR calculation provided by the *RNxQuest* package, using a single calculation based upon the combined pool of identifications from the four searches. Search results were then filtered for XLSMs with ld-scores (the summary score used by *xQuest*) where the FDR was <1%. Given that the calculation used for both approaches was the same, similar results were expected from both approaches, thereby validating the technical functionality of the reimplementation. Unique sets of detected modified amino acid positions identified for each cross-linking reagent, resulting from the two FDR analysis approaches, are compared in Figure 2b. The modified amino acid positions detected when using the different FDR analysis approaches were highly similar, with 94 unique cross-linked amino acid positions detected with the *RNxQuest* observed FDR calculation on the total pool versus 97 when using the separate *xProphet* runs for each Δm. The full redundant lists of XLSMs that result from the two FDR analysis approaches were compared, and the results are shown in Figure 2c. Of the XLSMs passing the 1% FDR threshold in both approaches, 97% of them overlap. The small number of XLSMs that are unique to each approach are explained by having ld-scores very close to the 1% FDR threshold score in their respective analysis pipeline, making them particularly sensitive to minor fluctuations in the ld-score filtering threshold that result from the two approaches. We therefore concluded that the TDC-based *RNxQuest* observed FDR calculation correctly replicates the monolink FDR calculation behavior of *xProphet*.

### Applying RNxQuest to CLIR-MS Cross-Linked Protein-RNA Samples

Having established that the *RNxQuest* analysis approach behaves as expected for a data set containing heterogeneous monolinks, we then tested the performance of the pipeline on three CLIR-MS protein–RNA cross-linking data sets, generated from three complexes with previously published structures. Using the outputs, we assessed the behavior of the pipeline on protein–RNA cross-linking data for which it is designed. The complexes are the FOX1 RRM with the FOX binding element (UGCAUGU), MBNL1 with its cognate binding sequence (CGCUU), and PTBP1 with a short polypyrimidine sequence (UCUCU). The corresponding data sets are described separately in more detail.^14^

**Figure 3:**
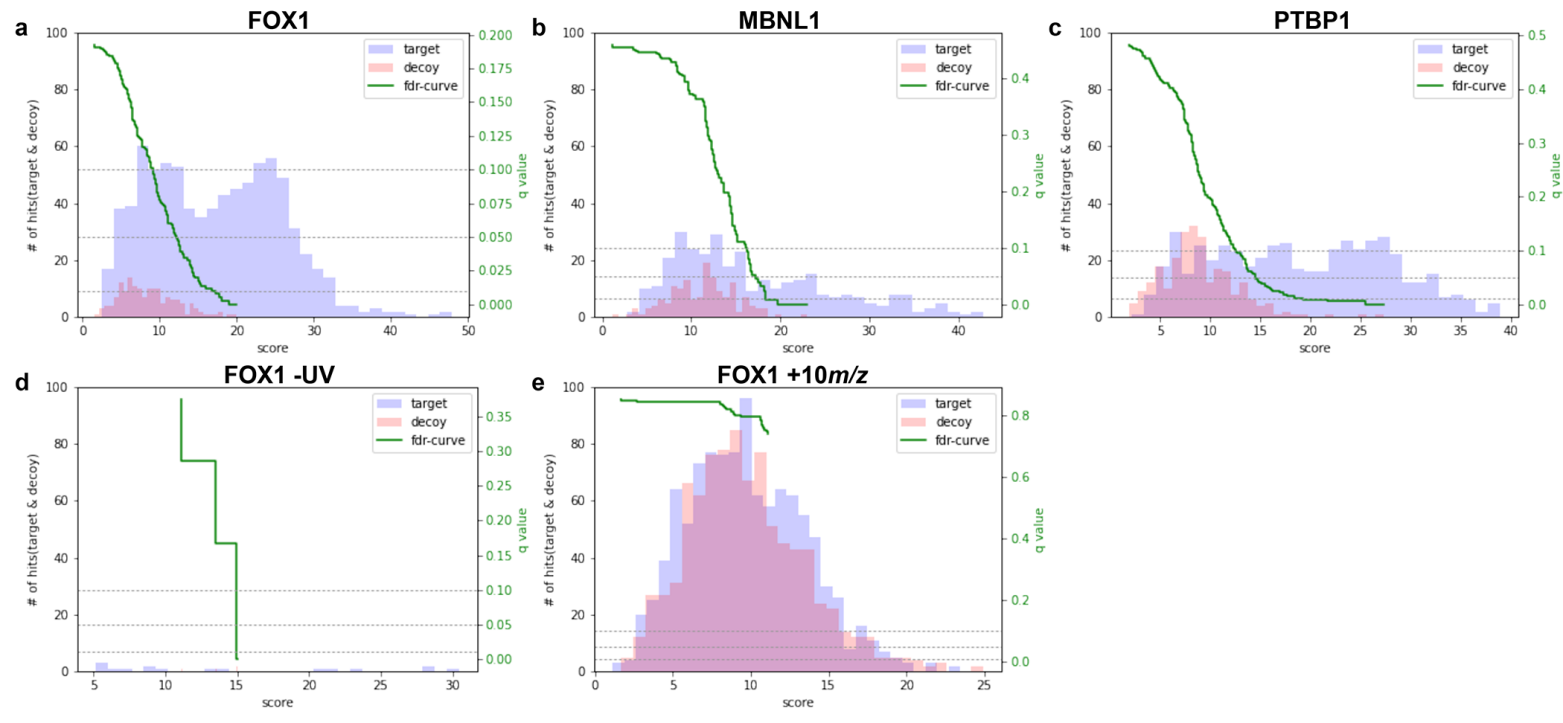
Evaluating the behavior of the *RNxQuest* observed FDR calculation for CLIR-MS data. (a–c) Target and decoy database match score distributions for CLIR-MS samples prepared with UV irradiated FOX1 (a), MBNL1 (b), and PTBP1 (c) protein–RNA complexes, respectively. For each distribution, a q-value is additionally calculated according to the *xQuest* ld-score. (d) Target and decoy database match score distributions for a FOX1 complex CLIR-MS sample prepared in the same way as panel a, except with UV irradiation omitted. (e) Target and decoy database match score distributions for a decoy data set, generated using the mzXML files produced for panel a, but shifting all *m*/*z* values by +10 *m*/*z* units.

Each data set was analyzed using the *RNxQuest* pipeline. For each analysis run, a protein database file containing the target protein sequence and a corresponding reversed and shuffled decoy sequence for that protein was used. FDR analysis was carried out using the *RNxQuest* observed FDR analysis function, and the score distributions of target and decoy database identifications for each complex were plotted (Figure 3a–c). From these distributions, a q-value curve is also calculated and plotted. The scores from all three protein–RNA complexes display a similar behavior. In each case, decoy database identifications (shown in red in each plot) form a single distribution at relatively low ld-scores, representing XLSMs by chance. The target identifications, shown in blue, segregate into two distributions; a lower scoring distribution overlapping with the decoy distribution, likely comprising mostly false-positive XLSMs by chance, and a higher scoring target distribution, which likely comprises true positive matches. In the case of the MBNL1 complex, the number of high scoring target database XLSMs is not so great. However, their clear segregation from the decoy identifications supports their acceptance as reliable identifications. Using a 1% FDR threshold, 1614, 374, and 654 XLSMs are returned for the FOX1, MBNL1, and PTBP1 complexes, respectively. Representative annotated spectra are shown in Figure S3. In summary, score distributions of target and decoy database sequence matches clearly segregate for all complexes. This suggests that a conventional TDC-based observed FDR calculation successfully helps control for false positive XLSMs in the novel isotope-labeled protein–RNA XL-MS data type.

Next, we more thoroughly investigated the possibility that any false positive XLSM could be assigned a high ld-score by the pipeline. We first analyzed a nonirradiated negative control sample, prepared using the same FOX1-FBE complex, including isotope-labeled RNA, as in Figure 3a. Sample processing and data analysis between this control sample and the sample in Figure 3a were identical, except for the omission of the UV irradiation step. Score distributions of target and decoy XLSMs made from this data set were once again plotted (Figure 3d). The nonirradiated data set exhibits overall very few putative XLSMs (against target or decoy databases) compared with the irradiated samples, even before FDR analysis. These have relatively low scores, in a range comparable to that of the decoy identifications in Figure 3a–c. Furthermore, the score distributions of target and decoy database matches almost exactly overlap, suggesting a high probability that all of these XLSMs match the database by chance. This is expected behavior, as a true identification requires both light and heavy isotopic species to be present in the mass spectra from which the identification is scored. In a CLIR-MS data set, light–heavy pairing depends on the presence of an RNA-derived modification to a peptide. In a nonirradiated sample, no such modifications are expected, and non-cross-linked RNA is expected to be lost during sample preparation, and therefore should not be present in the LC-MS/MS sample. The lack of substantial high-scoring identifications from the nonirradiated sample therefore suggests that the combination of light–heavy isotope pairing, and a TDC FDR approach, are a sufficiently stringent strategy to avoid high scoring false positive identifications.

Finally, to further ensure the robustness of the pipeline against high scoring false positive XLSMs, we tested the behavior of the pipeline on XLSMs from a known decoy data set. All peaks contained in mzXML files from the FOX1-FBE sample in Figure 3a were shifted by +10 *m*/*z* units, a principle demonstrated previously.^35^ In doing so, data are produced from which it is impossible by definition to produce a true positive identification. However, the data retain the features and qualities of a data set containing true-positive identifications. The decoy data set was then analyzed in the same way as the target data set in Figure 3a, and the score distribution was plotted (Figure 3e). In this case, both target and decoy database identifications each form overlapping low scoring distributions, which are remarkably similar. As with the nonirradiated sample, this is the expected behavior, as all XLSMs in this data set must be by chance, explaining the overlap between the distributions. Importantly, there is no high scoring target database XLSM distribution in this negative control data set, providing further evidence that the data analysis approach is specific enough to avoid artifacts. Taken together, the clear segregation of high-scoring true-positive distributions of target database matches from the false positive decoy database matches in target data sets (Figure 3a–c), and the lack of high scoring target identifications in control data sets (Figure 3d,e) suggests that the pipeline is both sensitive and sufficiently stringent when applied to CLIR-MS data.

**Figure 4:**
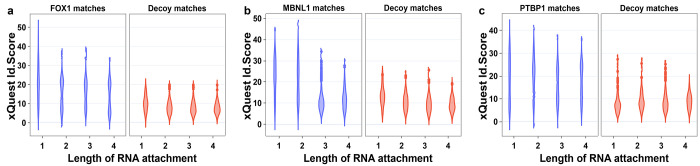
*xQuest* ld-score distributions may depend on the length of RNA adduct. (a–c) Score distributions for each of the complexes in Figure 3a–c presented as violin plots and additionally broken down by length of the RNA adduct.

When a protein–RNA cross-linking data set is produced using nuclease digestion, a variety of mono- and polynucleotide RNA adducts are present in the sample, attached to peptides. We speculated that because RNA tends to be more negatively charged than a peptide, different lengths of RNA attached to a peptide may have varying impacts on the fundamental behavior of the peptide–RNA adduct during mass spectrometric analysis. To investigate this possibility, we again plotted the score distributions for target and decoy identifications from the data sets shown in Figure 3a–c, but this time segregated by the length of RNA component of the peptide–RNA identification (Figure 4). We observed that across the three complexes, decoy identification score distributions do not seem to show much dependence on the length of RNA in the identification. However, in the cases of target identifications, longer RNA attachments to the peptides appear to correspond to lower scoring identifications. We questioned whether adopting a more sophisticated FDR control approach may be able to take advantage of these distinctive behaviors and therefore return a greater amount of unique structural information from each CLIR-MS data set, and in particular polynucleotide adducts which are useful for localizing cross-links within an RNA sequences.

### Assessment of mokapot as an Alternative FDR Analysis Strategy

Like most proteomics search engines for mass spectrometry data, identifications from *xQuest* are given a summary score (in this case the ld-score) that is calculated from a number of underlying subscores, describing the quality of the spectrum match to the database species using a fixed scoring model (described previously^19,20^). We hypothesized that the RNA length-dependent ld-score distributions could be founded upon subtle trends in output subscores, which are not prolific enough to be noticeable during manual interpretation. Machine learning approaches provide an alternative strategy to assess such underlying trends in high dimensional data sets such as this one. One such approach, the *Percolator* algorithm, has become a popular choice for proteomics data sets,^28,36^ and was recently reimplemented as part of a Python package, *mokapot*.^29^ The developers of the *mokapot* package propose that proteomics data sets with low abundance peptide modifications, such as protein–RNA XL-MS data sets, may especially benefit from a machine learning approach. To evaluate this possibility, FDR analysis for data sets shown in Figure 3a–c was repeated, using the *mokapot* package instead of the *RNxQuest* observed FDR calculation.

**Figure 5:**
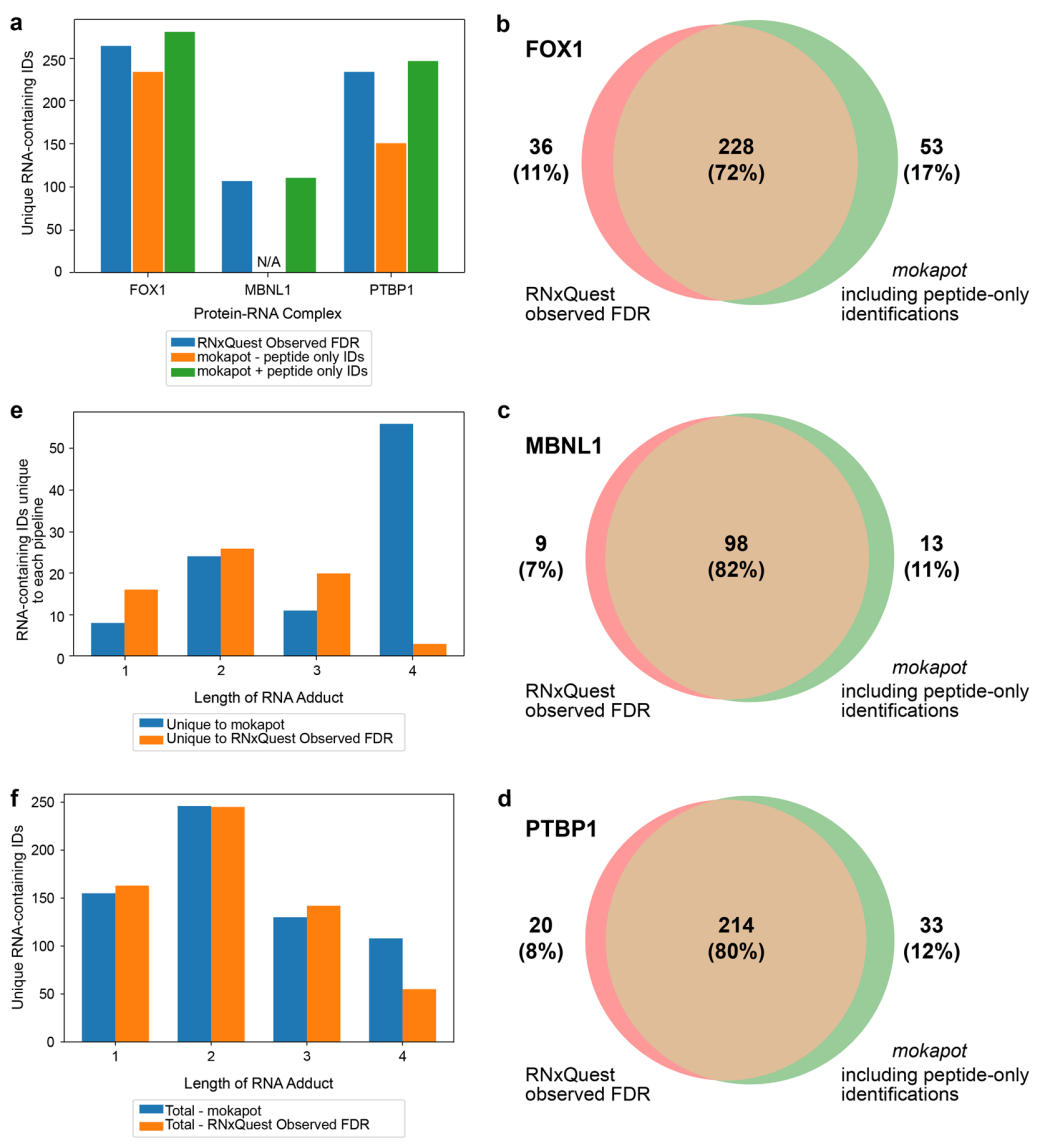
Comparing XLs identified when using *mokapot* for control of FDR versus the *RNxQuest* observed FDR calculation. (a) Numbers of unique identifications provided when the *RNxQuest* observed FDR approach, and *mokapot*, both in the presence and absence of peptide-only identifications, are used to control FDR in the FOX1, MBNL1, and PTBP1 protein–RNA XL-MS data sets. (b–d) Comparisons of unique protein–RNA identifications for *mokapot* analysis including peptide-only IDs, with the *RNxQuest* observed FDR calculation for the FOX1, MBNL1, and PTBP1 complexes, respectively. (e) Lengths of RNA adducts for uniquely identified peptide–RNA species across all complexes, when analyzed using the *mokapot* (including peptide-only identifications) package, compared with the *RNxQuest* observed FDR calculation. (f) Total numbers of unique protein–RNA IDs following *RNxQuest* observed FDR calculation and *mokapot* analysis, broken down by the length of the RNA adduct.

The number of XLSMs over a range of q-values assigned by *mokapot* is shown for each complex in Figure 5a. We filtered these results to achieve a nonredundant list of unique amino acid position and RNA adduct combinations, giving an overview of the unique structural information provided by this FDR analysis approach. This gave lists of 234 and 151 unique amino acid position–RNA adduct combinations for the FOX1 and PTBP1 data sets, respectively. For the MBNL1 data set, the execution repeatedly failed owing to the low number of input XLSMs. The initial results suggest that based on the same input data, *mokapot* provided fewer unique protein–RNA cross-links than the *RNxQuest* observed FDR approach.

It has been demonstrated previously that *mokapot* functions effectively on protein–RNA cross-linking data sets where a more complete range of identifications, such as those generated by an open modification search approach^16^ that includes linear unmodified peptides, is used as an input, instead of solely the RNA-modified peptides.^29^ In contrast, the default output of the *RNxQuest* search strategy contains only modified peptides, such as the results used in Figure 5a. This is due to the requirement for light-heavy isotope pairing, which is expected to occur only in RNA-containing species. The titanium dioxide enrichment step in the CLIR-MS protocol^9^ is not perfectly efficient, and some linear peptides are therefore still present in the LC-MS/MS sample, but are not included in the default search approach illustrated in Figure 1b. We hypothesized that including results from an additional peptide-only search in our inputs for the *mokapot* analysis could more effectively train the target-decoy discrimination model. By providing superior discrimination, additional unique amino acid plus RNA combinations could therefore potentially be identified.

To evaluate this possibility, we defined an additional parallel search of the scheme shown in Figure 1b to look for unmodified peptides using *xQuest*. We achieved this by defining a monolink mass of 0 Da, and adjusting the search parameters to look for “light-only” species, as described previously.^23^ A drawback of this approach is that identifications that are reliant or nonreliant upon isotope pairing may have different respective underlying subscoring properties. However, this limitation is here accepted for the lack of a more suitable alternative. We then executed the *mokapot* analysis for each complex using an input file consisting of the protein–RNA identifications used in the *RNxQuest* observed FDR calculation combined with the newly generated peptide-only identifications. Once again, we filtered the output identifications for unique combinations of amino acid positions and RNA adducts, excluding peptide-only identifications, which resulted in 281, 111, and 247 for the FOX1, MBNL1, and PTBP1 complexes, respectively. When compared with both the *mokapot* analysis that did not include peptide-only identifications and the *RNxQuest* observed FDR approach, the *mokapot* approach including peptide-only identifications results in the highest number of unique peptide–RNA combinations in all complexes (Figure 5a).

To investigate whether the *RNxQuest* observed FDR analysis approach and *mokapot* (including peptide-only identifications) yield distinct XL information, we compared the unique amino acid site and RNA modification combinations produced by each. Venn diagrams representing the overlap between the final identification lists produced by each approach are shown in Figure 5b–d, for the FOX1, MBNL1, and PTBP1 complexes, respectively. For all three protein–RNA complexes studied, around three-quarters of the unique identifications overlap between the *mokapot* FDR analysis approach and the simpler *RNxQuest* observed FDR calculation, with the *mokapot* approach yielding a greater number of unique identifications in each case. Where possible, cross-links were validated against published structures for both approaches (Tables S3, S4, and S5), with observed distances suggesting that detected XLs represent plausible protein–RNA contact sites. When unique identifications to each FDR analysis approach are broken down by the length of the RNA included in each identification, tetranucleotide RNA adducts are represented in the greatest number of unique identifications (Figure 5e), with 56 tetranucleotide identifications unique to the *mokapot* approach, compared with just 3 tetranucleotide identifications that are unique to the *RNxQuest* observed FDR calculation. The total number of unique tetranucleotide identifications returned across all 3 data sets is also substantially higher with *mokapot* (108) than with the *RNxQuest* observed FDR calculation (55, Figure 5f). This may suggest that the more sophisticated *mokapot* FDR control approach successfully recovers lower scoring polynucleotide identifications that are otherwise lost due to the RNA length-dependent scoring behavior shown in Figure 4a–c.

### Assessment of the Transferred FDR Analysis Strategy for CLIR-MS Data

In addition to the *mokapot* machine learning based approach, we implemented another approach termed “transferred FDR”.^30^ The transferred FDR approach was developed for more effective FDR estimation with low-abundance PTMs than can be achieved when all identifications are considered in the same pool. It assumes that in a sample containing many different types of low abundant PTMs, the FDR for each group of PTMs (separated into “bins”) will be distinct, and can be approximated for each bin using the proportion of decoy identifications which contain that PTM from the pool of all decoy identifications at a given score cutoff.^30,31^ Given the conceptual similarity between the heterogeneous RNA-derived monolinks identified in a CLIR-MS experiment and a set of heterogeneous PTMs in a more conventional proteomics experiment, we speculated that this approach might also be able to more effectively control the FDR where subsets of RNA modifications display distinct scoring behavior such as that shown in Figure 4a–c.

To evaluate this hypothesis, consolidated outputs from *RNxQuest* searches used in Figure 3a–c were divided into bins based on the sequence composition of the RNA component and subjected to the transferred FDR analysis (using a function included in the *RNxQuest* package). The resulting ld-score cutoff calculated for each bin is shown in Figure S4 for the FOX1, MBNL1, and PTBP1 complexes, respectively. In the majority of cases across all three data sets, there were insufficient numbers of decoy identifications for the linear approximation calculation component of the transferred FDR approach, resulting in a zero division error, and thus, the fallback strategy, using the *RNxQuest* observed FDR calculation, is used (described in the Experimental Section). Inclusion of peptide-only *xQuest* PSMs (subject to the same limitations acknowledged with respect to *mokapot* analysis above) did not lead to any increase in the number of bins for which a transferred FDR can be computed (Figure S4). This may be expected, given that their inclusion only modifies the total pool of decoys, and only adds an additional bin rather than changing the calculation for any of the existing bins.

To assess any unique information provided by the transferred FDR approach, the list of XLSMs passing the 1% transferred FDR threshold was refined to a unique list of amino acid positions and RNA modifications and compared with an equivalent list produced using the *RNxQuest* observed FDR calculation. Comparisons between the outputs from each approach are shown in Figure S4d–f for the FOX1, MBNL1, and PTBP1 complexes, respectively. The transferred FDR approach provides very little unique information compared with the *RNxQuest* observed FDR calculation, which is expected given that in most cases, the score assigned to each bin is anyway derived from the *RNxQuest* observed FDR calculation as a fallback approach. Taking an example, the PTBP1 complex has five unique identifications resulting from the transferred FDR approach, all of which result from the relatively low score threshold assigned to the mononucleotide “U” bin. However, these come at the cost of an overall lower number of unique identifications, compared with the *RNxQuest* observed FDR calculation. Based on the lack of uniform improvement in the number of unique identifications across all data sets, we conclude that the transferred FDR approach does not provide sufficient distinct information to warrant its routine use over the simpler *RNxQuest* observed FDR calculation. The relatively poor performance is likely explained by the low overall number of decoy identifications produced by the search strategy due to the light-heavy isotope-pairing requirement. This limitation is therefore specific to the nature of CLIR-MS data in their current form rather than a more general indication of the validity of the transferred FDR approach.

### Reanalysis of Previously Published Data Sets without Isotope-Labeled RNA

**Figure 6:**
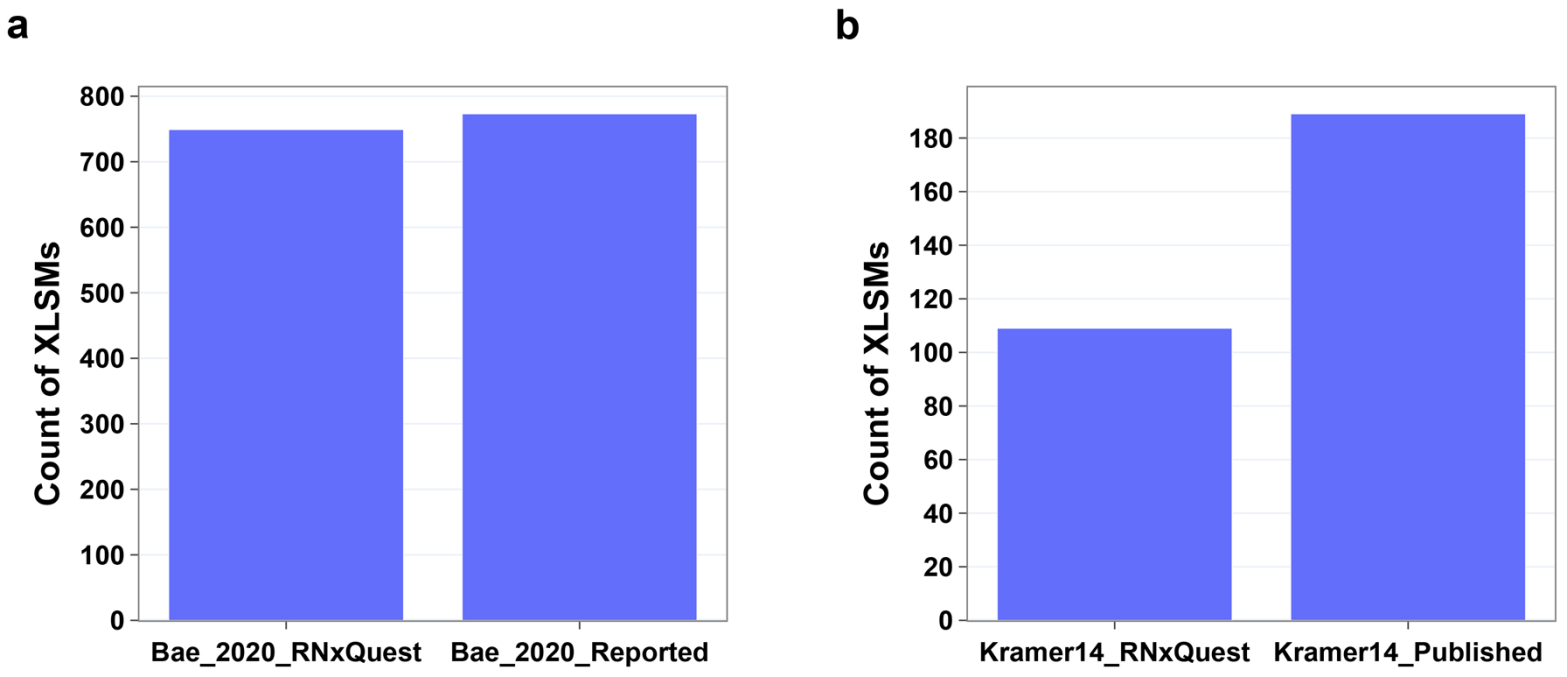
Reanalysis of previously published protein–RNA XL-MS data sets, where RNA is not isotopically labeled, using the *RNxQuest*-enabled approach. (a) XLSMs from spCas9-sgRNA with the *RNxQuest* approach compared with the MS-GF+ approach used by Bae et al. in their recent publication.^13^ (b) XLSMs from many human RNA-binding proteins cross-linked to their target RNAs^8^ with the *RNxQuest* approach. *RNxQuest* results are compared with RNP^xl^ approach.^11^

Thus, far, we have demonstrated that the *RNxQuest* package excels in its primary purpose as a dedicated software solution for the analysis of CLIR-MS data. However, while it may not always be the most appropriate choice in these cases, the *RNxQuest* pipeline additionally provides technical compatibility with protein–RNA XL-MS data sets produced without isotope-labeled RNA. To demonstrate this, we reanalyzed previously published data sets, and compared identifications made between the original publications with those outputted by our new analysis pipeline, using the *RNxQuest* observed FDR calculation approach. First, we analyzed a recently published data set containing a single protein, Cas9, UV cross-linked to a short guide RNA (sgRNA),^13^ which was originally analyzed using MS-GF+.^17^ When analyzed using the *RNxQuest* approach, 749 XLSMs are returned, compared to 773 in the originally published identification list (Figure 6a). Most notably, while the total number of XLSMs returned is lower with *RNxQuest*, in contrast with the original publication, a number of adducts other than mononucleotide Uare detected using *RNxQuest*. Representative annotated spectra from these cases are provided in Figure S5. Validation of cross-linked amino acid positions in these cases against the published structure suggests that these identifications likely represent biologically valid protein–RNA contact sites (Figure S6). Second, we selected an older but more complex data set, which includes many human RNA-binding proteins.^8^ With this more complex data set, the *RNxQuest* pipeline with the observed FDR approach returns a lower number of peptide–RNA identifications (109) than reported in the original publication (189). The difference could be explained by the larger number of possible products and, therefore, the larger search space considered for the *RNxQuest* search, which may increase the false discovery rate. Owing to technical differences between software packages, precisely matching analysis parameters between different pipelines is challenging. For completeness, further comparisons using permutations of ID and XLSM level FDR calculations and numerical comparisons are provided in Figure S7.

Taken together with the other data shown in this work, these examples demonstrate that the *RNxQuest* package provides a versatile solution for protein–RNA XL-MS data analysis. The package is technically capable of analysis of data from samples containing very large numbers of different protein–RNA complexes; however, other packages may be more appropriate for this sample type. Rather, *RNxQuest* excels most in the task for which it is designed, in identification of peptide–RNA cross-links in purified cross-linked protein–RNA samples. For data produced from purified protein–RNA complexes, the package returns plentiful FDR-controlled XLSMs, both with assistance from and in absence of isotope-labeled RNA, and in the latter case may also return complementary XL identifications compared with other software packages.

## Discussion

Here we introduce a software solution for analysis of protein–RNA XL-MS data, focusing on low complexity samples (such as purified complexes) where stable isotope-labeled RNA is used during complex formation, as per the CLIR-MS approach.^9^ The particular challenges related to this data type are derived from the low abundance of target species present in the sample, itself a problem due to the low efficiency of the cross-linking reaction. Analysis of this data type therefore requires a careful balance between sensitivity and stringency, to maximize identifications made from a data set while maintaining confidence in those identifications.

The RNA-length dependent shifts in the score distribution of identifications made with the *RNxQuest* pipeline may initially suggest that longer RNA modifications always lead to lower quality identifications. The difference in scoring behavior may be explained by the high relative weight of the total ion current (TIC) value in the *xQuest* scoring function.^20^ It has previously been observed that the RNA component of a peptide–RNA adduct will also fragment under HCD conditions, leading to RNA-derived marker ions.^8^ Unlike RNPxl,^12^ the *RNxQuest* search strategy does not make use of these marker ions, as the formation of such ions may be dependent on factors such as mass, charge state or collision energy threshold for peptide fragmentation. Instead, light-heavy isotope labeling is used to ensure the peptide modification is RNA-derived. This leaves marker ions as unassigned peaks in the spectrum, subsequently reducing the *xQuest* TIC subscore. We therefore conclude that this behavior is expected based on the functionality of the software and does not reduce the overall reliability of longer RNA adducts.

Of the more sophisticated FDR analysis approaches investigated in this work, the *mokapot* machine-learning approach appears to show promise. In this study, when peptide-only identifications are included, many unique identifications are made when the *mokapot* analysis is used compared with the simpler observed *RNxQuest* FDR calculation. This suggests that routinely generating a bespoke scoring model or each data set based on such an algorithm could prove a useful strategy to maximize the information retrieved from a CLIR-MS data set. The developers of the *mokapot* package suggest that an ideal minimum number of identifications upon which to train a machine learning model such as this is around 5,000.^37^ The nature of CLIR-MS identifications made by *xQuest*, such as requiring both light and heavy isotopic variants of a species to be present and only searching for peptides with an attached RNA modification, means that this threshold will tend to not be met when using *xQuest*-identified modified peptides alone as an input for *mokapot*. We attempt to bring the number of XLSMs used as an input closer to this value using peptide-only identifications. However, the practical feasibility of this strategy as a routine solution might be limited. First, the computational efficiency of the isotope labeling strategy is lost. For isotope-labeled identification searches, only spectra that contain the expected delta mass shift need to be scored against the database. For a peptide-only search, the parameters do not include the light-heavy isotope pairing requirement, and therefore all spectra must be searched, resulting in vastly extended processing times. Furthermore, in an isotope-labeled search, *xQuest* merges the peaks of a pair of spectra for identification scoring,^19^ whereas a light-only search leads to identifications being scored against a single spectrum. This means the underlying nature of subscores of the XLSMs may differ from those of the peptide-only spectrum matches, especially in cases where isotope-enabled search is used. It has also been previously suggested that adding identifications, such as unmodified peptides, which are not relevant to the experimental hypothesis (i.e., protein–RNA interactions) may adversely affect how effectively the FDR in a set of identifications is estimated.^38^

Despite its relative technical simplicity compared with the *mokapot* approach, the conventional target-decoy discrimination provided by the *RNxQuest* observed FDR calculation appears to provide effective differentiation between target database matches by chance and those likely to represent the presence of the assigned peptide–RNA species. When compared with the *mokapot* approach without the input of peptide-only identifications, the *RNxQuest* observed FDR approach even yields a greater number of unique peptide–RNA identifications. However, the strong overlap of the majority of unique identifications between outputs of the *RNxQuest* observed FDR calculation and *mokapot* analysis serves as an orthogonal metric of their quality, suggesting that this core overlapping group of identifications resulting from both approaches are reliable. In addition, the cross-links were mapped on existing structures, with calculated distances in line with previous observations.

In summary, the data analysis approach for protein–RNA XL-MS data presented here must strike a balance between sensitivity and specificity, given the underlying properties of this data type. The sensitivity requirement is achieved by employing stable isotope labeling in the CLIR-MS approach, combined with the *xQuest* software for data analysis. This approach facilitates an effective signal-to-noise ratio enhancement by spectral merging prior to scoring. The work presented here explores multiple strategies to ensure specificity of the approach. We demonstrate that a conventional TDC based FDR calculation executed on a pool of heterogeneous monolink-like identifications produced by a CLIR-MS experiment successfully discriminates between target database matches that are likely by chance and those that likely represent the presence of the species in the sample. We further report that a machine-learning based FDR analysis approach based on the *mokapot* implementation of the *Percolator* algorithm also achieves this aim, while additionally producing a slightly greater number of unique identifications when peptide-only identifications are included as inputs. However, the number of XLSMs used for *mokapot* analysis here does not meet the minimum number suggested number by the developers, risking overfitting the model to a specific data set. We therefore propose a conventional TDC FDR analysis approach as the default method used in analysis of CLIR-MS data, and that the *mokapot* analysis could provide a useful alternative in cases where the data set under consideration contains sufficient numbers of identifications upon which to train the model.

## Data Availability

All *RN**xQuest* outputs have been deposited to the ProteomeXchange Consortium (http://proteomecentral.proteomexchange.org) via the PRIDE^34^ partner repository with the data set identifier PXD039754. Raw data for the model protein–RNA complexes have been deposited to the same repository with data set identifier PXD029930. Raw data for the Cas9 and human RBP complex data sets were downloaded from the PRIDE repository using data set identifiers PXD016254 and PXD000513, respectively.

## Supporting Information Available

Figure S1 explains the available FDR control strategies and XLSM filtering approaches employed in *RNxQuest*; Figure S2 illustrates example outputs from the *RNxQuest* analysis pipeline; Figure S3 shows representative example annotated spectra as identified by *RNxQuest* from the FOX1 complex; Figure S4 displays summary outputs from *RNxQuest* from the FOX1, MBNL1, and PTBP1 protein–RNA XL-MS data sets using the transferred FDR approach; Figure S5 shows representative example annotated spectra, from XLs identified in the Cas9-sgRNA data; Figure S6 illustrates that nonmononucleotide-U XLSMs identified by *RNxQuest* represent plausible protein–RNA contact sites in the published structure of this complex; Figure S7 provides additional representations of underlying data from Figure 6 using permutations of ID and XLSM level FDR calculation and numerical comparison; Table S2 contains the list of *xQuest* search output features used as inputs for *mokapot* (PDF)
Table S1 contains summary outputs of XLSM monolinks identified after cross-linking BSA, using either *xProphet* or the *RNxQuest* observed FDR calculation for FDR control (XLSX)
Table S3 provides summary outputs of protein–RNA XLSMs identified from the FOX1 complex, using *RNxQuest* and the observed FDR, *mokapot*, and transferred FDR approaches, as well as measured cross-link distances (XLSX)
Table S4 provides summary outputs of protein–RNA XLSMs identified from the MBNL1 complex, using *RNxQuest* and the observed FDR, *mokapot*, and transferred FDR approaches, as well as measured cross-link distances (XLSX)
Table S5 provides summary outputs of protein–RNA XLSMs identified from the PTBP1 complex, using *RNxQuest* and the observed FDR, *mokapot*, and transferred FDR approaches, as well as measured cross-link distances (XLSX)
Table S6 contains summary outputs of protein–RNA XLSMs identified after reanalysis of previously published Cas9 and human RBP data sets using *RNxQuest* and the observed FDR approach (XLSX)
