# Supplementary material for: RNxQuest: An Extension to the xQuest Pipeline Enabling Analysis of Protein–RNA Cross-Linking/Mass Spectrometry Data
Source: J Proteome Res. 2023 Sep 5;22(10):3368–82. doi: 10.1021/acs.jproteome.3c00341 (PMC10563164; doi:10.1021/acs.jproteome.3c00341)
Supplement: Supplementary file 1 — pr3c00341_si_001.pdf [file pr3c00341_si_001.pdf]

Supporting Information to

RNxQuest: an extension to the *xQuest* pipeline enabling analysis of protein-RNA cross-linking/mass spectrometry data

Chris P. Sarnowski[1,2]; Michael Götze[1,3]; Alexander Leitner[1,*]

[1] Institute of Molecular Systems Biology, Department of Biology, ETH Zürich, 8093 Zurich, Switzerland

[2] Systems Biology PhD Program, University of Zürich and ETH Zürich, Zurich, Switzerland

[3] Current address: Institute of Organic Chemistry, Department of Biology, Chemistry and Pharmacy, FU Berlin, 14195 Berlin, Germany

* Corresponding author (Email: leitner@imsb.biol.ethz.ch, phone: +41 44 633 2698)

Table of Contents

Figure S1. FDR control strategies in *RNxQuest*. S-2

Figure S2. Examples of JuPyter Notebook output. S-3

Figure S3. Annotated spectra for FOX1-UGCAUGU. S-4

Figure S4. Comparison of different methods for FDR calculation in *RNxQuest*. S-5

Figure S5. Annotated spectra for Cas9-sgRNA. S-6

Figure S6. Visualization of non-U cross-links on Cas9-sgRNA. S-8

Figure S7. Additional ways of calculating FDRs on the Bae et al. and Kramer et al. datasets. S-9

Table S2. Features for *RNxQuest* / *mokapot* analysis. S-10

Summary of additional tables, references S-12

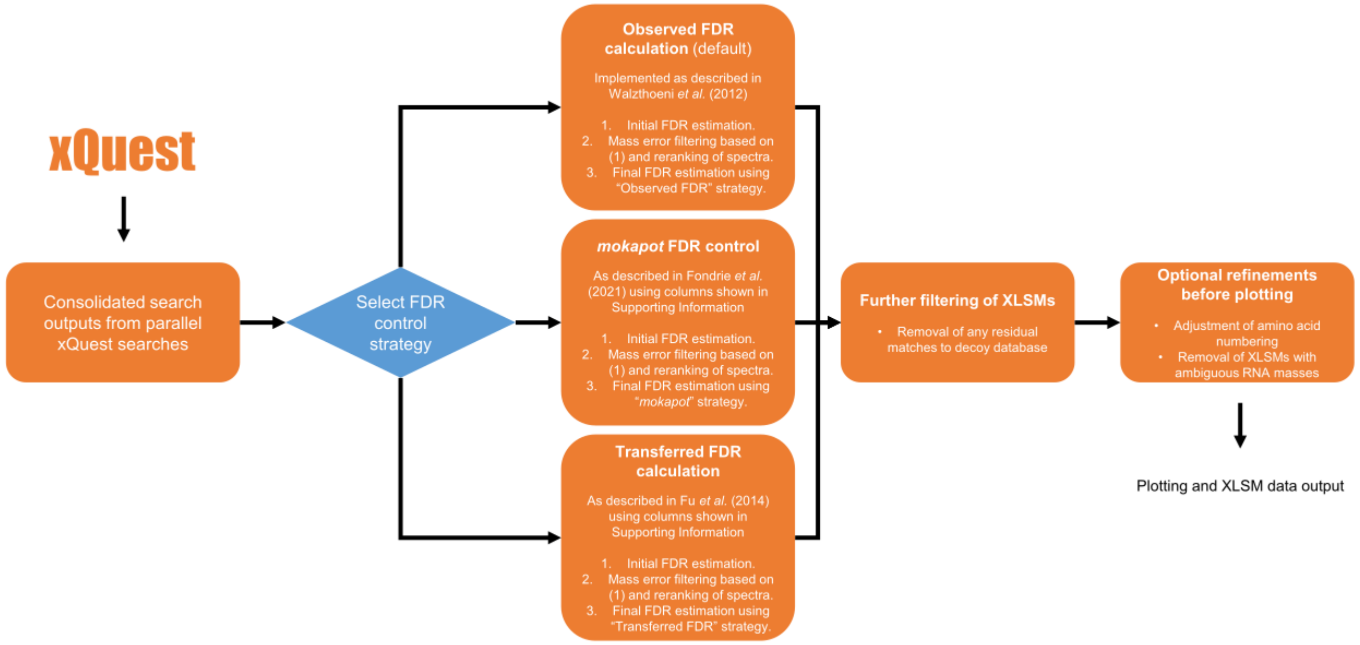

Supporting Information Figure S1

Multiple FDR control strategies are available as functions in *RNxQuest*. These strategies, along with the filtering applied after FDR analysis, are outlined in the flowchart above.

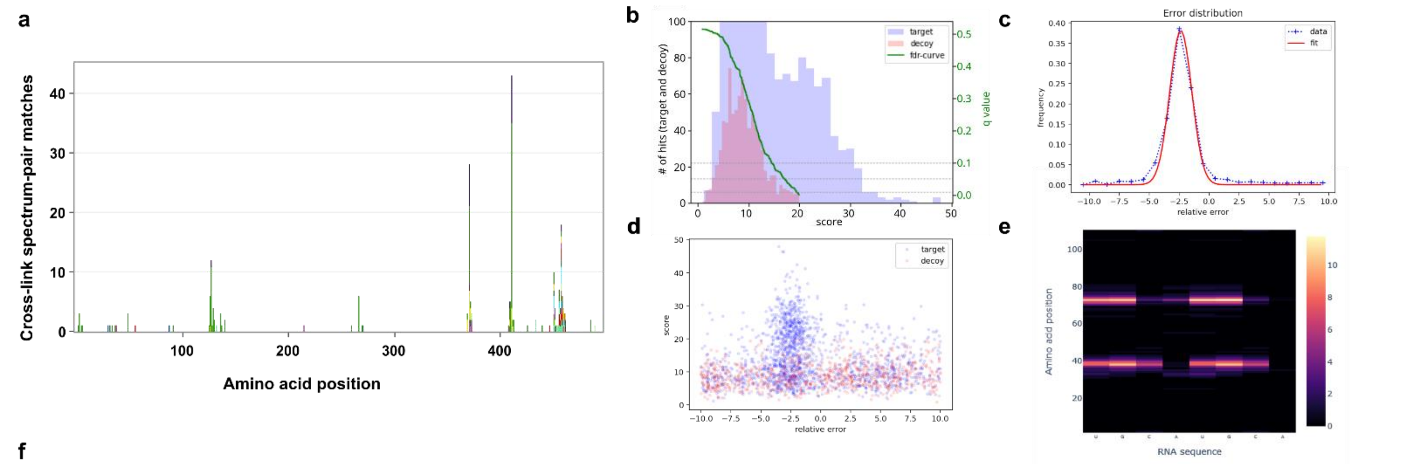

f

Id	Protein	Light_spectrum	Heavy_spectrum	AbsPos1	RNA_Mr	RNA_composition
GQPIYIQFSNHK-Y5-U	db PROT01 PTBP1	210217_LU02_csarnowski_2102_2b.c.09911.09911.2	210217_LU02_csarnowski_2102_2b.c.09692.09692.2	130	324.036	U
NFQIPPSVSEEDLK-L14-CU	db PROT01 PTBP1	210217_LU02_csarnowski_2102_2b.c.26553.26553.3	210217_LU02_csarnowski_2102_2b.c.26588.26588.3	461	627.062	CU
GQPIYIQFSNHK-Y5-U	db PROT01 PTBP1	210217_LU02_csarnowski_2102_2a.c.09994.09994.3	210217_LU02_csarnowski_2102_2a.c.09747.09747.3	130	324.036	U
…	…	…	…	…	…	…

Neutral_Loss	Isobaric_RNA_Composition	Mr_Calculated	Mr_measured	Mz	z	Error_rel_ppm	TIC	ld-score	FDR_score	FDR_type	FDR_level
Natural_	U Natural_	1754.76652	1754.763525	878.3895874	2	-1.7	0.17764	38.95	0	Observed FDR	id
Natural_-H2	CU Natural_-H2	3620.57495	3620.566369	1207.863281	3	-2.4	0.05638	38.31	0	Observed FDR	id
Natural_	U Natural_	1754.76652	1754.762292	585.9285889	3	-2.4	0.17968	38.3	0	Observed FDR	id

Supporting Information Figure S2

Generic examples of outputs from the JuPyter Notebook based post-processing pipeline produced with each *RNxQuest* execution. (a) Bar plot, illustrating the number and sequence composition of cross-linked RNA adducts detected at a given amino acid position in a protein. (b) XLSM score distributions for target and decoy database matches. (c) Error distribution of target database identifications, represented as a histogram. The distribution is used for mass error filtering. (d) Scatter plot of mass error deviation versus XLSM score. (e) Heatmap representation of inferred position of cross-links within the RNA sequence specified in the sample. (f) Example output from the summary .csv file outputted from the analysis. This format also represents a recommended data reporting format for this data type.

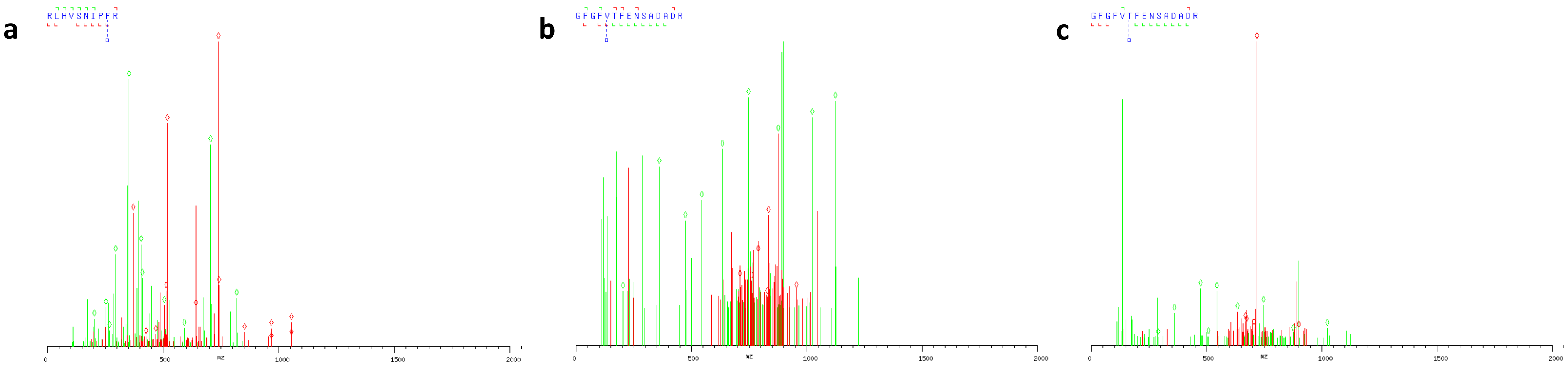

RLHVSNIPFR + U, $[M+3H]^{3+}$

common ions m/z	rel. int.	fragment	xlink ions m/z	rel. int.	fragment
204.2791	8.9	b3 2+	644.3041	12.2	y2 1+
253.7520	12.6	b4 2+	741.2671	100.0	y3 1+
270.1941	5.1	b2 1+	854.3360	4.5	y4 1+
297.2501	30.1	b5 2+	968.4270	5.7	y5 1+
354.2751	87.5	b6 2+	1055.4370	7.8	y6 1+
407.2900	33.3	b3 1+	371.2071	43.7	y3 2+
410.8311	22.3	b7 2+	427.5301	3.0	y4 2+
506.3170	13.3	b4 1+	468.6481	3.9	y9 3+
593.3110	6.0	b5 1+	515.1921	18.0	b10 3+
707.3521	66.1	b6 1+	521.0181	73.0	y10 3+
820.4840	15.7	b7 1+			

GFGFVTFENSADADR + CGU, $[M+3H]^{3+}$

common ions m/z	rel. int.	fragment	xlink ions m/z	rel. int.	fragment
205.1051	12.0	b4 2+	834.8701	29.0	b7 2+
361.2010	39.9	y3 1+	956.4421	12.2	b9 2+
476.1811	27.8	y4 1+	761.9280	14.4	y12 3+
547.2750	32.5	y5 1+	713.0161	14.8	y11 3+
634.3361	43.9	y6 1+	761.9280	14.4	y12 3+
748.2870	55.5	y7 1+	791.2621	20.4	b14 3+
877.4141	47.4	y8 1+	830.5341	10.8	y14 3+
1024.4150	51.0	y9 1+			
1125.5021	54.6	y10 1+			

GFGFVTFENSADADR + AGUU, $[M+4H]^{4+}$

common ions m/z	rel. int.	fragment	xlink ions m/z	rel. int.	fragment
290.1020	2.5	y2 1+	900.6501	4.9	b14 3+
361.1340	10.6	y3 1+	668.5950	7.7	y13 4+
476.2331	18.6	y4 1+	675.7010	6.7	b14 4+
508.2891	2.8	b5 1+	705.4501	5.6	y14 4+
547.3080	17.6	y5 1+	719.5500	100.0	y15 4+
634.3871	10.9	y6 1+			
748.2881	13.0	y7 1+			
877.2910	3.6	y8 1+			
1024.3900	5.5	y9 1+			

Supporting Information Figure S3

Representative annotated spectra from the FOX1-UGCAUGU protein-RNA complex, with identifications returned by *RNxQuest* using the *RNxQuest* observed FDR calculation. Spectra shown are the preprocessed spectra as searched by *xQuest*, following the merging of spectra derived from light and heavy precursors, for cross-links RLHVSNIPFR-F9-U, GFGFVTFENSADADR-V5-CGU, and GFGFVTFENSADADR-T6-AGUU, in panels (a), (b), and (c), respectively. Peak assignments are shown on the bottom, with observed mass-to-charge ratio ([m/z]), intensity relative to base peak in % (rel. int.), and fragment ion type and charge state (fragment). Assignments are either designated as “common” (same m/z in light and heavy spectrum, shown in green in the spectrum) or “xlink” fragments (containing the RNA modification and therefore showing a mass shift between light and heavy spectrum, shown in red in the spectrum).

a

	Score cut for 1% FDR +Peptide only IDs	Score cut for 1% FDR -Peptide only IDs
A	18.98	16.86
AC	18.98	16.86
ACG	18.98	16.86
ACGU	18.98	16.86
ACU	18.98	16.86
AGU	18.98	16.86
AGUU	17.2	17.2
AU	18.08	18.08
C	18.98	16.86
CG	18.98	17.67
CGU	12.51	12.51
G	18.98	16.86
GU	19.97	19.97
GUU	12.11	12.11
Peptide	1.74	N/A
U	18.98	16.86

b

	Score cut for 1% FDR +Peptide only IDs	Score cut for 1% FDR -Peptide only IDs
C	15.63	18.35
CCG	22.53	18.35
CCGU	22.53	16.02
CG	22.53	18.35
CGU	22.53	18.35
CGUU	22.53	18.35
CU	22.53	18.35
CUU	22.53	18.35
G	22.53	18.35
Peptide	7.35	N/A
U	22.53	18.27
UU	23.04	23.04

c

	Score cut for 1% FDR +Peptide only IDs	Score cut for 1% FDR -Peptide only IDs
C	19.54	19.27
CCU	19.54	19.27
CCUU	19.54	19.27
CU	19.54	25.53
CUU	19.54	19.27
Peptide	4.84	N/A
U	11.19	11.19

Tables Legend
- RNxQuest Observed FDR
- Transferred FDR

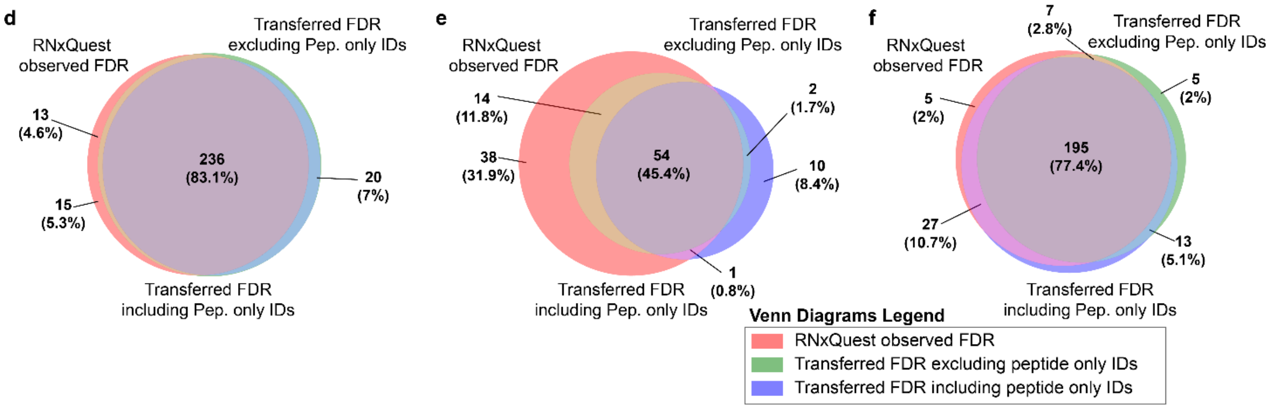

Supporting Information Figure S4

Comparison of different methods for FDR calculation. (a)-(c) FDR scores for each bin using transferred FDR calculations for FOX1, MBNL1, and PTBP1, respectively. Few bins contain enough identifications to technically permit calculation of transferred FDR, and those that do have similar score to the *RNxQuest* observed FDR. Performance seems to decrease with inclusion of peptide-only IDs. (d)-(f) Overlap in unique IDs (i.e. AbsPos1 + mod_mass combinations) compared between transferred FDR and *RNxQuest* observed FDR. Transferred FDR seldom yields any new IDs, whilst with the MBNL1 complex, also eliminating a large number of identifications permitted by the observed FDR calculation.

a

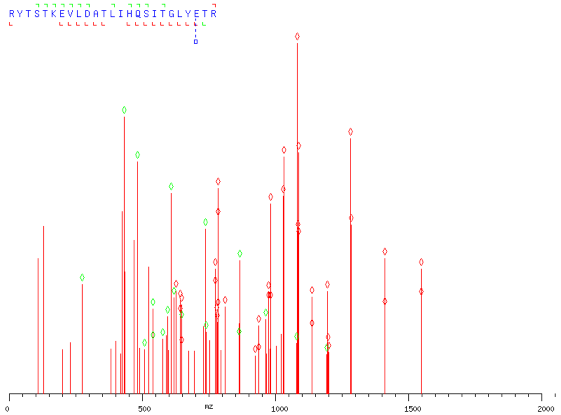

RYTSTKEVLDATLIHQSITGLY<u>E</u>TR + U, [M+4H]$^{4+}$

m/z	rel. int.	fragment	m/z	rel. int.	fragment
276.1668	31.1	y2 1+	812.3445	24.7	y4 1+
433.7229	78.9	b7 2+	864.9667	15.8	b15 2+
483.2579	66.2	b8 2+	866.4396	37.8	b7 1+
508.2526	12.6	b4 1+	925.4312	10.8	y5 1+
539.7997	24.0	b9 2+	938.4870	11.3	y14 2+
540.3022	14.8	b19 4+	965.4994	21.0	b8 1+
576.9819	15.5	b15 3+	973.9982	26.0	y15 2+
597.3153	21.9	b10 2+	982.4498	54.1	y6 1+
609.3017	57.1	b5 1+	1031.5106	56.4	y16 2+
619.6693	27.3	b16 3+	1079.5582	14.4	b19 2+
626.3271	29.2	y14 3+	1083.4968	100.0	y7 1+
642.6755	22.3	y9 2+	1088.0522	44.4	y17 2+
648.6809	20.4	b17 3+	1137.5884	18.0	y18 2+
649.0147	25.2	y3 1+	1193.6145	11.1	b10 1+
737.3962	46.8	b6 1+	1196.5814	29.0	y8 1+
739.8991	17.5	b13 2+	1202.6083	11.7	y19 2+
774.8702	35.5	y11 2+	1283.6136	72.7	y9 1+
781.4021	22.5	b25 4+	1411.6735	38.4	y10 1+
785.6553	24.0	y25 4+	1548.7310	35.4	y11 1+

b

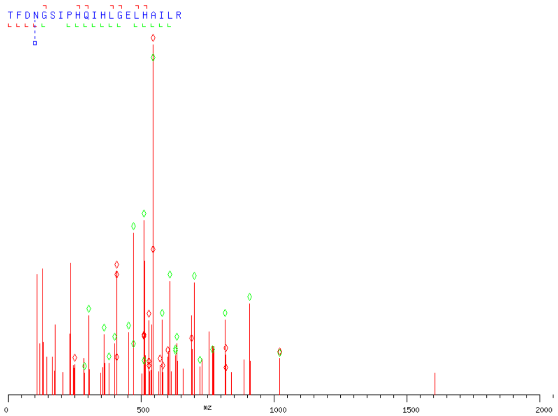

TFD<u>N</u>GSIPHQIHLGELHAILR + G, [M+5H]$^{5+}$

m/z	rel. int.	fragment	m/z	rel. int.	fragment
251.1513	8.5	b9 5+	579.8443	21.4	y10 2+
288.2035	6.1	y2 1+	581.8028	6.3	b13 3+
305.1967	22.5	y5 2+	601.3978	10.7	b14 3+
361.7382	17.1	y6 2+	609.3861	32.2	y5 1+
379.2115	8.9	y17 5+	630.8306	10.5	y17 3+
401.2902	14.6	y3 1+	636.3839	14.5	y11 2+
409.2385	32.2	b16 5+	691.4055	14.1	b10 2+
454.7716	17.6	y8 2+	700.4153	31.9	y12 2+
472.3275	46.1	y4 1+	722.4622	7.9	y6 1+
473.3292	12.6	y17 4+	768.9501	10.9	y13 2+
511.3149	15.0	b16 4+	817.4652	21.4	y14 2+
512.9654	49.6	y13 3+	818.4642	5.8	b5 1+
531.1022	7.5	y21 5+	908.5297	26.0	y8 1+
545.3165	94.3	y14 3+	1021.614	9.9	y9 1+
545.6503	100.0	b17 4+	1022.623	10.3	b16 2+
573.3043	8.3	y18 4+			

c

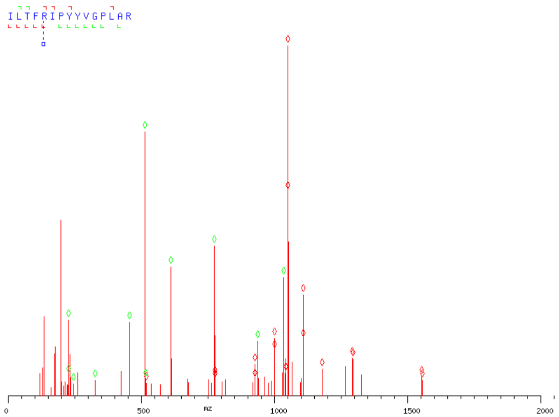

ILTF<u>R</u>IPYYVGPLAR + UU, [M+3H]$^{3+}$

m/z	rel. int.	fragment	m/z	rel. int.	fragment
227.1754	21.4	b2 1+	1042.977	6.4	b13 2+
228.6511	5.8	y4 2+	1052.486	100.0	y13 2+
246.1563	3.3	y2 1+	1109.028	28.8	y14 2+
328.2243	4.4	b3 1+	1181.492	7.6	b5 1+
456.294	20.9	y4 1+	1295.579	10.3	b6 1+
513.315	75.3	y5 1+	1555.693	4.3	b8 1+
518.284	4.4	y9 2+			
518.7846	3.6	b8 3+			
612.3836	36.7	y6 1+			
775.4467	42.6	y7 1+			
777.214	4.4	y15 3+			
927.922	4.4	y11 2+			
938.5054	15.5	y8 1+			
1001.459	12.7	y12 2+			
1035.561	33.6	y9 1+			

(continued on next page)

d

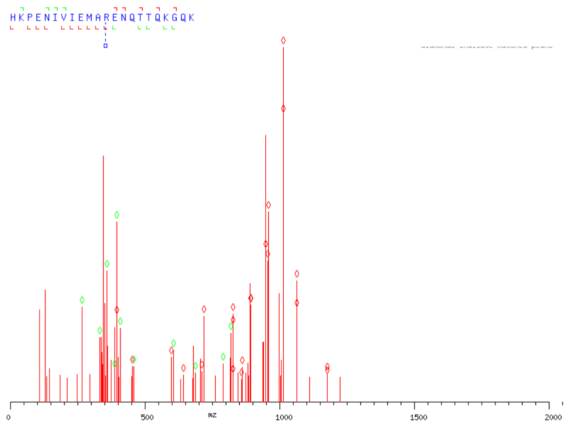

HKPENIVIEMARENQTTQKGQK + A, $[M+4H]^{4+}$

m/z	rel. int.	fragment	m/z	rel. int.	fragment
266.1612	26.8	b2 1+	790.4421	10.6	y7 1+
332.1936	18.2	y3 1+	818.4434	19.4	b7 1+
360.1962	36.8	b6 2+	827.408	24.7	y12 2+
387.7215	8.8	y10 3+	828.4091	7.2	y19 3+
395.733	50.7	y7 2+	860.7667	9.6	y20 3+
396.2349	24.0	y11 4+	892.9284	27.2	y13 2+
409.7312	20.7	b7 2+	948.9673	42.5	y22 3+
454.2311	9.9	b13 4+	957.9531	53.5	y14 2+
460.2894	9.8	y4 1+	1014.496	100.0	y15 2+
597.4101	12.5	b18 4+	1064.03	34.0	y16 2+
606.3006	14.4	b5 1+	1177.596	8.0	y18 2+
643.3655	7.5	b14 3+			
689.3928	8.1	y6 1+			
712.1161	8.5	y22 4+			
719.3853	24.0	b16 3+			

e

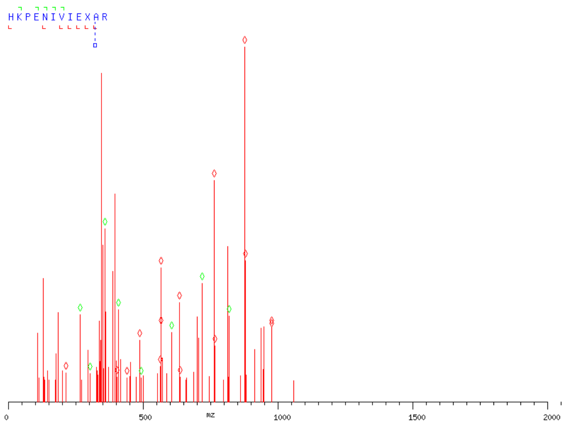

HKPENIVIEM^{ox}AR + CGU, $[M+3H]^{3+}$

m/z	rel. int.	fragment
213.1604	8.2	y3 3+
266.1618	24.6	b2 1+
303.6529	7.8	b5 2+
360.1971	48.6	b6 2+
402.7376	6.9	y8 3+
409.7315	25.9	b7 2+
439.7068	6.7	y5 2+
489.243	17.3	y2 1+
492.2554	6.7	b4 1+
565.8292	20.9	y12 3+
606.2999	19.4	b5 1+
636.2799	27.9	y3 1+
719.3841	33.4	b6 1+
765.3219	62.2	y4 1+
818.4528	24.0	b7 1+
878.4066	100.0	y5 1+
977.4781	20.9	y6 1+

Supporting Information Figure S5

Representative annotated spectra from the Cas9-sgRNA protein-RNA complex, with identifications returned by *RNxQuest* using the *RNxQuest* observed FDR calculation. In the absence of isotope labelling, spectra shown are single scans for cross-links RYTSTKEVLDATLIHQSITGLYETR-E23-U (a), TFDNGSIPHQIHLGELHAILR-N4-G (b), ILTFRIPYYVGPLAR-R5-UU (c), HKPENIVIEMARENQTTQKGQK-R12-A (d), and HKPENIVIEM^{ox}AR-A11-C (e), respectively. The annotated spectra shown are as searched by *xQuest* after preprocessing (removal of low intensity peaks). Peak assignments are shown on the bottom, with observed mass-to-charge ratio ([m/z]), intensity relative to base peak in % (rel. int.), and fragment ion type and charge state (fragment).

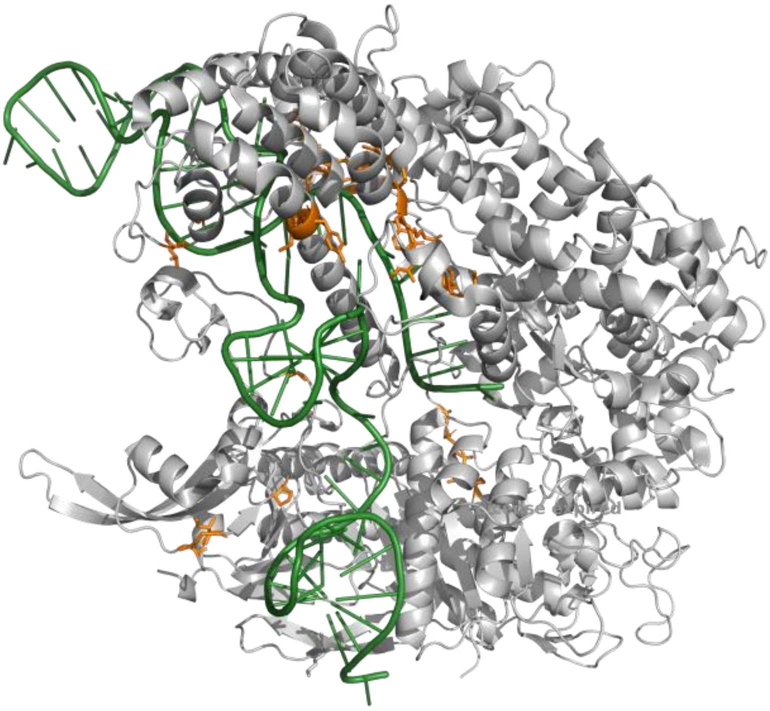

Supporting Information Figure S6

In contrast to the XLSMs originally reported with the data, when *RNxQuest* is used to search the previously published Cas9 complex data, a number of non-U RNA adducts are returned with the results. The length of the RNA used in the original analysis, and the highly redundant nature of ribonucleotide sequences, makes validation of the RNA component of the cross-link more challenging than with the short oligonucleotides used for the FOX1, MBNL1 and PTBP1 complexes described in the main article. However, the amino acid positions of non-mononucleotide-U XLs detected by *RNxQuest* can be validated with more certainty, and are colored orange on the published structure for this complex[1] (PDB 4ZT0). Cursory analysis reveals the proximity of all of these amino acids to the RNA chain (colored green), suggesting the validity of these complementary identifications.

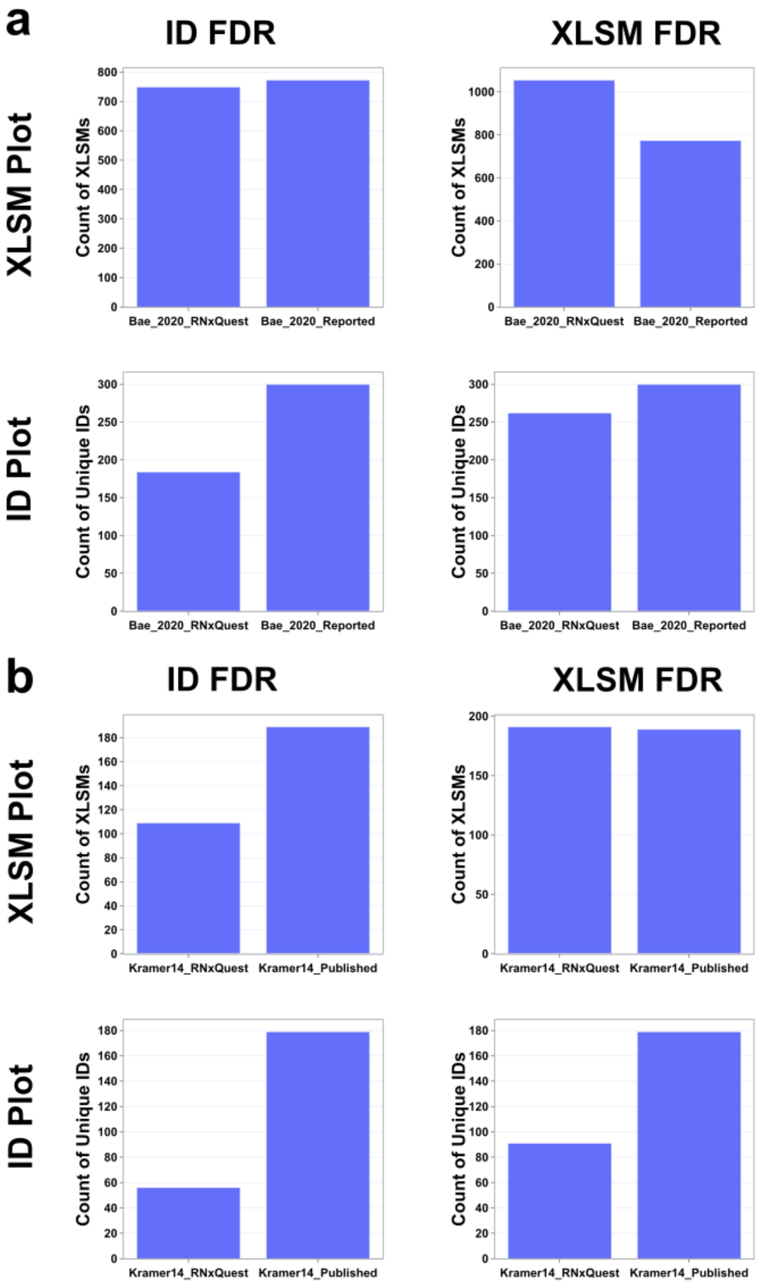

Supporting Information Figure S7

In the main article, the performance of *RNxQuest* is compared with other search engines used to search non-isotope-labelled protein-RNA XL-MS data. In **Figure 6** in the main article, the outputs from the *RNxQuest* observed FDR calculation, using ID-level FDR, and counting the outputted redundant XLSMs outputted, are presented. However, differences between the precise functionalities of search software makes direct comparison challenging. For completeness, additional analyses using XLSM-level FDR, and plotting the returned identifications both at XLSM level, and ID level (i.e. redundant identifications of the same cross-link in different spectra are counted only once) are shown here.

Supporting Information Table S2

Data sub-scores and features from *RNxQuest* results used as input for FDR analysis using the *mokapot* package. These features are produced when using the *RNxQuest* to PIN converter function provided in the *RNxQuest* package. Further information about requirements for a PIN file can be found on the *Percolator* repository wiki (https://github.com/percolator/percolator/wiki/Interface#tab-delimited-file-format).

**Feature name**	**Description and justification**	**Justification**
SpecId	Concatenation of xQuest “Id” column, plus scan number and label from below.	Required by mokapot
Label	1 for target identifications and -1 for decoy identifications	Required by mokapot
ScanNr	Concatenation of light and heavy scan numbers, each padded to 7 digits with leading zeroes.	Required by mokapot
Mr	Calculated mass of identification (provided in xQuest output)	Aids determination of match to database
Mr_precursor	Observed mass of identification (provided in xQuest output)	
apriori_pmatch_common	Probability of database match based on common ions*	Included in default xQuest scoring function
apriori_pmatch_xlink	Probability of database match based on xlink ions*	Included in default xQuest scoring function
wTIC	Weighted total ion current – the proportion of ion intensity in the spectrum explained by expected fragment species	Included in default xQuest scoring function
AbsPos1	Amino acid position number relative to the FASTA file	Represents localisation of RNA modifications on the whole protein
AA	Single letter code of amino acid encoded as an integer, where A=1, C=3, etc	All amino acid types can form XLs to RNA, but some may be favoured
mod_mass	Calculated mass of RNA modification	Facilitates stratification of identifications by RNA modification type

Search	Delta mass shift of the search from which the identification derives (rounded to 2 decimal places)	Aids the user in understanding which parameters were used to generate the identification.
xcorrb	Correlation between theoretical and observed common fragment ions	Included in default xQuest scoring function
xcorrx	Correlation between theoretical and observed xlink fragment ions	Included in default xQuest scoring function
match_odds	Probability of XLSM	Included in default xQuest scoring function
intsum	Sum of assigned ion intensities	Included in default xQuest scoring function
error_rel	Relative error of identification measured Mr to calculated	Useful reference field to aid further refining of results.
error	Absolute error of identification measured Mr to calculated	Useful reference field to aid further refining of results.
RNA_<n>	One column per length of RNA adduct permitted in the search, i.e. with a maximum RNA length of 2, there would be 2 columns of this format, called RNA_1 and RNA_2. The value is set to 1 or 0 dependent on if the length of RNA adduct in the XLSM is equal to n.	Facilitates stratification of identifications by RNA modification length
rel_pos	Relative position of RNA adduct on peptide from NTD (0) to CTD (1)	Relative position of RNA may impact fragmentation properties of peptide-RNA ion
Peptide	Id column from xQuest output	Required by mokapot
Proteins	Database entry name from which identification is made	Required by mokapot

*Terminology according to the xQuest software package[2].

Tables provided separately

Table S1: Summary outputs of mono-links PSMs identified after cross-linking BSA, using either xProphet or the *RNxQuest* observed FDR calculation for FDR control (XLSX).

Table S3: Summary outputs of protein-RNA XLSMs identified from the FOX1 complex, using *RNxQuest* and the observed FDR, mokapot, and transferred FDR approaches, as well as measured cross-link distances (XLSX).

Table S4: Summary outputs of protein-RNA XLSMs identified from the MBNL1 complex, using *RNxQuest* and the observed FDR, mokapot, and transferred FDR approaches, as well as measured cross-link distances (XLSX).

Table S5: Summary outputs of protein-RNA XLSMs identified from the PTBP1 complex, using *RNxQuest* and the observed FDR, mokapot, and transferred FDR approaches, as well as measured cross-link distances (XLSX).

Table S6: Summary outputs of protein-RNA XLSMs identified after reanalysis of previously published Cas9 and human RBP datasets using *RNxQuest* and the observed FDR approach (XLSX).

References for Supporting Information

(1) Jiang, F.; Zhou, K.; Ma, L.; Gressel, S.; Doudna, J. A. A Cas9–Guide RNA Complex Preorganized for Target DNA Recognition. *Science* **2015**, *348* (6242), 1477–1481. https://doi.org/10.1126/science.aab1452.

(2) Rinner, O.; Seebacher, J.; Walzthoeni, T.; Mueller, L. N.; Beck, M.; Schmidt, A.; Mueller, M.; Aebersold, R. Identification of Cross-Linked Peptides from Large Sequence Databases. *Nat. Methods* **2008**, *5* (4), 315–318. https://doi.org/10.1038/nmeth.1192.
